# The lncRNA *SLNCR* Recruits the Androgen Receptor to EGR1-Bound Genes in Melanoma and Inhibits Expression of Tumor Suppressor p21

**DOI:** 10.1016/j.celrep.2019.04.101

**Published:** 2019-05-21

**Authors:** Karyn Schmidt, Johanna S. Carroll, Elaine Yee, Dolly D. Thomas, Leon Wert-Lamas, Steven C. Neier, Gloria Sheynkman, Justin Ritz, Carl D. Novina

**Affiliations:** 1 Department of Cancer Immunology and Virology, Dana-Farber Cancer Institute, Boston, MA 02215, USA; 2 Department of Medicine, Harvard Medical School, Boston, MA 02215, USA; 3 Broad Institute of Harvard and MIT, Cambridge, MA 02141, USA; 4 Center for Cancer Systems Biology (CCSB) and Department of Cancer Biology, Dana-Farber Cancer Institute, Boston, MA 02215, USA; 5 Department of Genetics, Harvard Medical School, Boston, MA 02115, USA; 6 Harvard TH Chan School of Public Health, Boston, MA 02115, USA; 7 Present address: Alnylam Pharmaceuticals, Cambridge, MA, USA; 8 Lead Contact

## Abstract

Melanoma is the deadliest form of skin cancer, affecting men more frequently and severely than women. Although recent studies suggest that differences in activity of the androgen receptor (AR) underlie the observed sex bias, little is known about AR activity in melanoma. Here we show that AR and EGR1 bind to the long non-coding RNA *SLNCR* and increase melanoma proliferation through coordinated transcriptional regulation of several growth-regulatory genes. ChIP-seq reveals that ligand-free AR is enriched on *SLNCR*-regulated melanoma genes and that AR genomic occupancy significantly overlaps with EGR1 at consensus EGR1 binding sites. We present a model in which *SLNCR* recruits AR to EGR1-bound genomic loci and switches EGR1-mediated transcriptional activation to repression of the tumor suppressor p21^Waf1/Cip1^. Our data implicate the regulatory triad of *SLNCR*, AR, and EGR1 in promoting oncogenesis and may help explain why men have a higher incidence of and more rapidly progressive melanomas compared with women.

## In Brief

Long non-coding RNA function can be understood by defining their interacting proteins. Schmidt et al. demonstrate that the melanoma lncRNA SLNCR binds to AR and complexes with different transcription factors to mediate invasion or proliferation. Thus, lncRNAs regulate distinct transcriptional programs based on specific protein interactions.

## Graphical Abstract

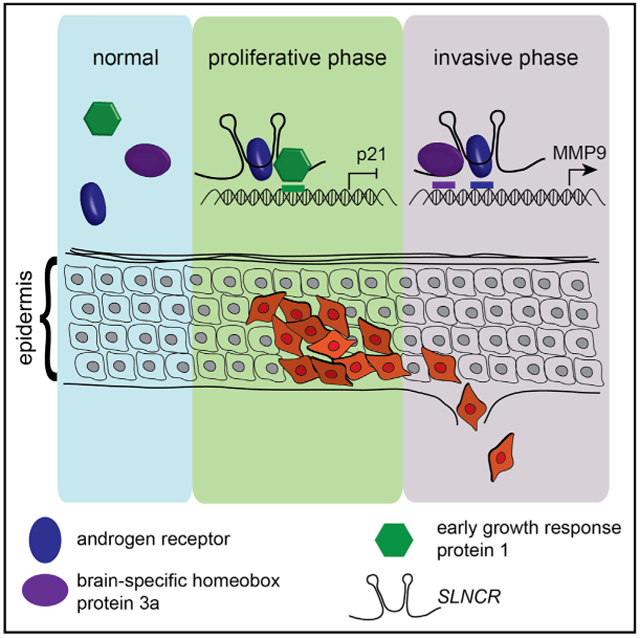

## INTRODUCTION

The worldwide incidence of melanoma has been on the rise for the past 30 years. In the United States, there are ~73,000 new cases diagnosed and ~10,000 deaths annually attributed to melanoma (Siegel et al., 2015). Of these deaths, approximately two-thirds occur in males, the result of a well-established sex bias disfavoring males in melanoma etiology (Bidoli et al., 2012; de Vries et al., 2007; Fisher and Geller, 2013; Geller et al., 2002; Joosse et al., 2011; Schwartz et al., 2002; Swetter et al., 2009). In addition to a significant survival advantage compared with males (38%), females demonstrate fewer metastases, a longer delay before relapse, and higher curable rates, strongly suggesting a biological basis for the observed sex bias (Gamba et al., 2013; Joosse et al., 2011,^2012^).

The androgen receptor (AR) regulates tumorigenesis in many human cancers, including prostate, breast, kidney, lung, bladder, and liver cancer (Chang et al., 2014). AR may function as a tumor suppressor or oncogene, likely dependent on cellular context and the presence or absence of AR-modulating factors. For example, AR activity in prostate cancer cells may be modulated by RNAs, including the long non-coding RNA (lncRNA) *HOTAIR* (Zhang et al., 2015), or by proteins, such as the transcriptional repressor RE1-silencing transcription factor (REST; also called neuron-restrictive silencer factor [NRSF]) (Svensson et al., 2014). Early studies suggested that AR has oncogenic functions in melanoma and that differences in AR function or expression might explain the observed melanoma gender differences (de Vries et al., 2008; Joosse et al., 2011; Li et al., 2013b; Micheli et al., 2009; Morvillo et al., 1995, 2002; Spanogle et al., 2010). In direct support of an oncogenic function for AR, we recently showed that AR increases melanoma invasion through transcriptional upregulation of the matrix metalloproteinase MMP9 (Schmidt et al., 2016). Interestingly, this regulation occurs independently of canonical AR activation, in which an androgen (such as testosterone) binds to the receptor to elicit downstream transcriptional patterns. Instead, AR-mediated invasion requires a novel lncRNA, *SLNCR*; specifically, the abundant *SLNCR1* isoform that directly binds to and recruits AR to the *MMP9* promoter.

lncRNAs are transcripts of more than 200 nt that lack an open reading frame and exhibit cell type and tissue-specific expression. lncRNAs are important regulators of tissue physiology and disease processes and may function either as oncogenes or tumor suppressors (Li and Chen, 2013; Serviss et al., 2014). Although the fundamental mechanism of many lncRNAs remains unknown, many (like *SLNCR*) function through direct interactions with proteins. Using a highly sensitive technique developed for the identification of RNA-associated transcription factors called RATA (RNA-associated transcription factor array), we showed that *SLNCR* binds to multiple transcription factors, possibly regulating their downstream transcriptional activities (Schmidt et al., 2016, 2017). In addition to AR and Brn3a, both of which are required for *SLNCR1*-mediated regulation of *MMP9*, we also identified early growth response 1 (EGR1) as a candidate *SLNCR1*-interacting transcription factor (Schmidt et al., 2016).

EGR1 is a zinc-finger transcription factor implicated in many human cancers, likely functioning as a tumor suppressor. In prostate cancer, EGR1 is a critical regulator of AR function (Yang and Abdulkadir, 2003). In melanoma, EGR1 has been implicated in apoptosis, cell growth, and fibronectin matrix synthesis (Ahmed et al., 1996; Gaggioli et al., 2005; Muthukkumar et al., 1995; Sells et al., 1995). Other roles for EGR1 in melanomagenesis, including biological consequences of possible physical interactions with *SLNCR* and/or AR, have not been described.

Because *SLNCR* imparts oncogenic function to AR in the absence of canonical androgen-induced signaling, we sought a more complete mechanistic understanding of AR function as a possible explanation for the observed melanoma sex bias. Here we examine ligand-independent, *SLNCR*-regulated AR function in melanoma and find that AR directly binds many *SLNCR*-regulated genes. Our data suggest that *SLNCR* recruits AR directly to EGR1-bound chromatin. AR and *SLNCR* appear to act as a transcriptional switch, reversing EGR1-mediated upregulation of the p21^Waf1/Cip1^ tumor suppressor gene. These results suggest that *SLNCR*, AR, and EGR1 form a novel regulatory triad that regulates melanoma proliferation. These data demonstrate that a comprehensive evaluation of AR function in melanoma is critical for understanding the mechanistic underpinnings of the melanoma sex bias.

## RESULTS

### *SLNCR* Isoforms Exhibit Both Unique and Overlapping Functions

Melanomas express at least 3 isoforms of *SLNCR*, *SLNCR1*–*SLNCR*3 (Figure S1A). *SLNCR1* is the shortest and most prevalent isoform, whereas *SLNCR2* and *SLNCR3* differ only in the inclusion of an additional exon of varying length. We demonstrated previously that *SLNCR1* binds to AR and recruits it to the MMP9 promoter and that *SLNCR1* and AR are required for transcriptionally upregulating *MMP9* expression and promoting melanoma invasion (Schmidt et al., 2016). Surprisingly, unlike *SLNCR1*, neither *SLNCR2* nor *SLNCR3* upregulate *MMP9* or increase melanoma invasion, suggesting that *SLNCR* isoforms have at least partially unique functions (Figures S1B and S1C). Isoform-specific functions cannot be explained by differences in AR binding because all 3 isoforms contain the RNA region required for AR binding and consistently bind AR *in vivo* (Figures S1A and S1D). Thus, all three *SLNCR* isoforms likely regulate AR activity.

**Figure 1.**
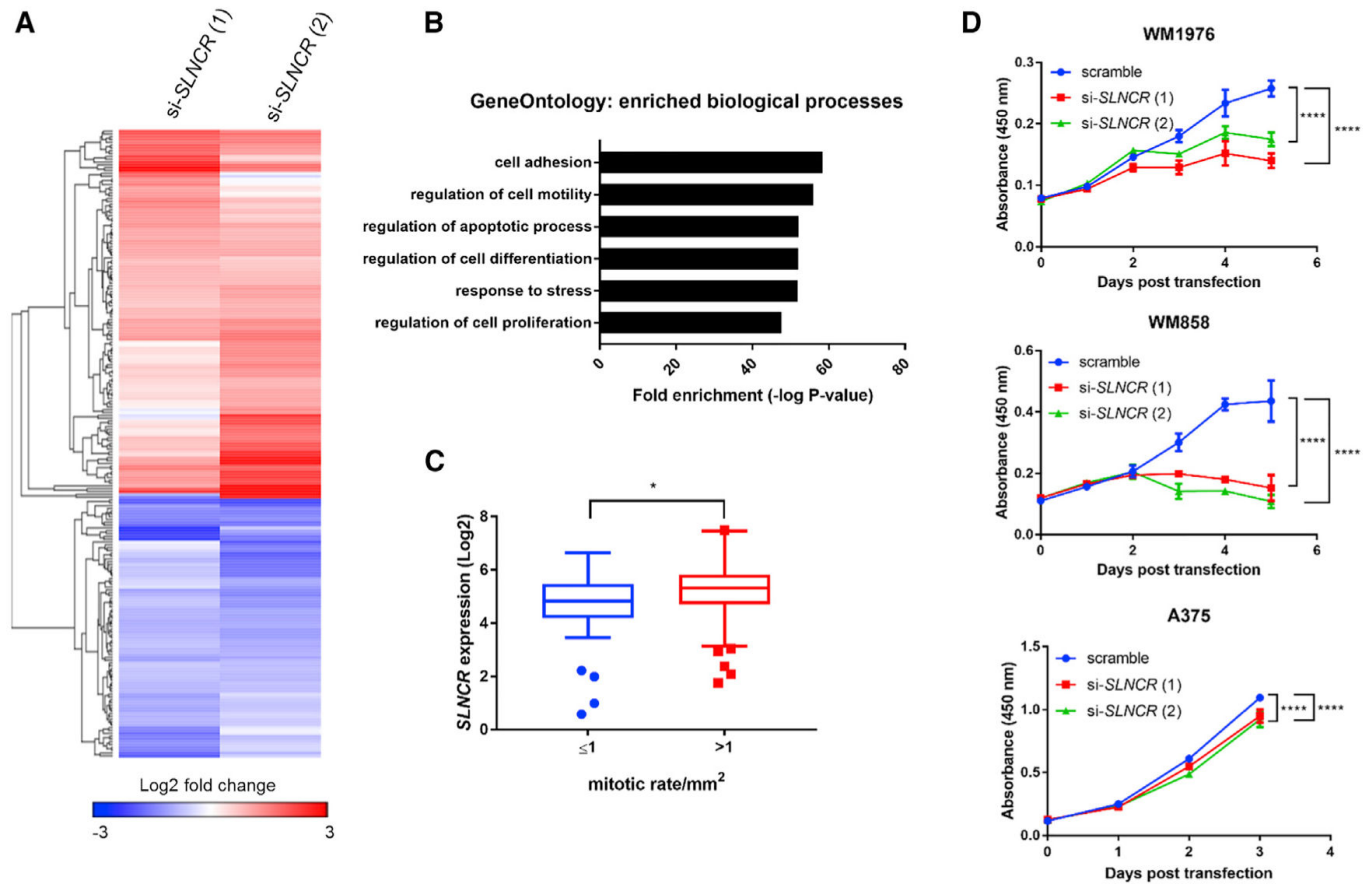
*SLNCR* Regulates Melanoma Proliferation (A) Heatmap of differentially expressed genes upon knockdown of *SLNCR* in MSTC WM1976. Shading represents the log2 fold change compared with the scramble siRNA control. Genes are clustered with Euclidean distance and average linkage clustering. (B) Gene Ontology (GO) biological process enriched in the differentially expressed genes represented in (A). (C) Tukey boxplots of *SLNCR* expression in TCGA melanomas (n = 172) exhibiting low(≤ 1)or high (>1) primary mitotic growth rates. Significance was calculated using a Mann-Whitney test: *p < 0.05. (D) The indicated MSTCs were seeded in 96-well plates and transfected with the indicated siRNAs, and cell proliferation was quantified at the indicated time points using WST-1 proliferation reagent. Each assay was repeated 2–3 times, and one representative assay is shown. Error bars represent the mean ± SD of 3 technical replicates. Significance was calculated using two-way ANOVA with Dunnett test for multiple comparisons testing. ****p < 0.0001. See also Figures S1 and S2.

To investigate the role of isoform-overlapping *SLNCR* function in melanoma development, we designed small interfering RNAs (siRNAs) to knock down all *SLNCR* isoforms (hereafter, *SLNCR* refers to *SLNCR1*–*SLNCR3*) (Figure S1A). These siRNAs knocked down *SLNCR* by ~60%–80% in two patient-derived melanoma short-term cultures (MSTCs), WM1976 and WM858, and by 50%–70% in the immortalized malignant melanoma cell line A375 (Figure S2A). Importantly, MSTCs have undergone minimal passages outside of the patient and provide an accurate genetic model of melanoma (Lin et al., 2008). WM1976 and WM858 exhibit moderate to high levels of *SLNCR* expression and are amenable to genetic studies requiring transfection of DNA or RNA (Schmidt et al., 2016). Using RNA sequencing (RNA-seq), we transcriptionally profiled melanoma cells before and after siRNA-mediated knockdown of *SLNCR* in WM1976, the MSTC expressing the highest levels of *SLNCR*. Depletion of *SLNCR* significantly dysregulated 222 genes compared with a scramble siRNA control, upregulating 131 genes and downregulating 91 genes (p < 0.01; Figure 1A; Table S1).

Analysis of the full melanoma dataset from The Cancer Genome Atlas (TCGA) revealed that *SLNCR* expression is significantly correlated with expression of 120 candidate target genes (p < 0.05; Table S1; Schmidt et al., 2016). Moreover, expression of *SLNCR* and 62 of these target genes is significantly correlated, even when correcting for multiple-hypothesis testing (Bonferroni correction, p < 0.00023), strongly suggesting that our RNA-seq analysis faithfully identified many *SLNCR*-regulated genes.

We next compared *SLNCR* differentially expressed genes (DEGs) to isoform-specific *SLNCR1* DEGs identified previously (Schmidt et al., 2016). Of the 222 *SLNCR* DEGs, 41 genes (18.5%) were also dysregulated upon knockdown of *SLNCR1* (Table S1; p < 0.01 in both RNA-seq experiments). Moreover, the majority of *SLNCR* and *SLNCR1* DEGs (35 of 41) displayed concordant dysregulation (Pearson r = 0.27, p < 0.0001; Figure S2B), reflecting that *SLNCR1* and *SLNCR* similarly regulate these genes.

Importantly, 6 DEGs are discordantly regulated upon *SLNCR* or *SLNCR1* knockdown, supporting our conclusion that *SLNCR* isoforms have unique functions. Two of these discordantly regulated genes, fibronection (*FN1*) and integrin subunit beta-1 (*ITGB1*), regulate cell matrix adhesion (Gene Ontology [GO] enrichment analysis, GO category 0007161, p = 1.02 × 10^−06^, false discovery rate = 1.58 × 10^−02^), whereas another two of these genes, transmembrane protein 45A (TMEM45A) and Ras-related protein RAB31, have been implicated in cancer invasion and cell adhesion (Grismayer et al., 2012; Guo et al., 2015). Combined with the findings that (1) *SLNCR1*, but not *SLNCR2* or *SLNCR3*, increases melanoma invasion (Figure S1) and (2) knockdown of *SLNCR1*, but not *SLNCR*, significantly decreases *MMP9* (Table S1; data not shown), we conclude that *SLNCR* isoforms uniquely regulate melanoma invasion. Because knockdown of *SLNCR* or *SLNCR1* only similarly dysregulates the majority of DEGs, and all three *SLNCR* isoforms bind AR, we focused on isoform-overlapping regulation of AR.

### *SLNCR* and Ligand-Independent AR Increase Melanoma Cell Proliferation

GO Enrichment analysis of the 222 DEGs upon *SLNCR* knockdown identifies significant enrichment of genes involved in multiple cancer-relevant processes, including cell adhesion and/or motility, apoptosis, differentiation, response to stress, and proliferation (Figure 1B). We were particularly interested in possible roles of *SLNCR* in melanoma proliferation because *SLNCR* has been implicated in proliferation of pancreatic, gastric, and non-small-cell lung cancers (Huang et al., 2017; Lu et al., 2017b; Shi et al., 2016; Zheng et al., 2016). Consistent with enrichment of cell proliferation genes identified with GO analysis (~22% or 49 of 222 DEGs, p = 2.54 × 10^−48^; Figure 1B), interrogation of TCGA data reveals that *SLNCR* expression is significantly correlated with the mitotic growth rate of primary melanomas (Spearman r = 0.20, p = 0.0083; Figure S2C), with slow-growing melanomas expressing significantly lower levels of *SLNCR* (mitotic growth rate ≤ 1 versus > 1 mitosis/mm^2^, p = 0.014; Figure 1C). *SLNCR* knockdown significantly decreased proliferation of both MSTCs compared with a scramble siRNA control (p < 0.0001; Figure 1D). Proliferation of the melanoma cell line A375 was slightly reduced upon *SLNCR* knockdown (p < 0.0001), likely because of a lower endogenous levels of *SLNCR*, which limits the fold depletion and range of knockdown-related phenotypes (Figure 1D; Schmidt et al., 2016). Importantly, knockdown of *SLNCR* did not alter the percentage of apoptotic cells in either MSTCs or in A375 melanoma cells (Figure S2D). Collectively, these experiments indicate that *SLNCR* increases melanoma proliferation. Because depleting *SLNCR1* alone did not affect cell growth, *SLNCR* isoforms likely share an overlapping role in regulation of melanoma proliferation, with *SLNCR2* and *SLNCR3* able to functionally compensate in the absence of *SLNCR1* (Schmidt et al., 2016).

**Figure 2.**
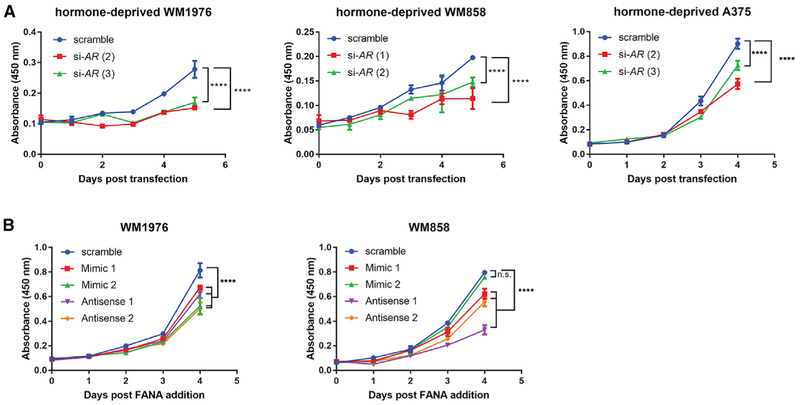
*SLNCR* and AR Cooperatively Regulate Melanoma Proliferation in a Hormone-Independent Manner The indicated cells were either transfected with the indicated siRNAs (A) or 2′-FANA-modified oligos were added to the cell medium (B) 24 h after cells were seeded in 96-well plates. Cell proliferation was quantified using WST-1 reagent, as in Figure 1D. Error bars represent the mean ± SD of 3 technical replicates. Significance was calculated using two-way ANOVA with Dunnett test for multiple comparisons testing. n.s., not significant; ****p < 0.0001. See also Figure S3.

Consistent with previous studies implicating AR in melanoma cell proliferation (Morvillo et al., 1995, 2002), the anti-androgen flutamide, which competes with androgen for binding to AR, significantly decreased melanoma cell proliferation (p < 0.0001; Figure S3A). Although this suggests an androgen-dependent role of AR in melanoma proliferation, *SLNCR* and AR interact even in the absence of canonical ligand-induced AR activation (Schmidt et al., 2016). Although standard cell culture conditions use fetal bovine serum, which contains exogenous hormones, it is unknown whether these standard cell culture conditions accurately reflect the natural hormone state of the melanoma tumor microenvironment. To test whether AR regulates melanoma proliferation in the absence of androgens, we quantified melanoma cell proliferation in hormone-depleted medium (phenol-red free medium supplemented with charcoal-stripped medium) before and after AR depletion. Two AR-targeting siRNAs resulted in ~60%–90% of knockdown of AR in 3 melanoma cells (Figures S3B and S3C). AR knockdown significantly decreased proliferation of hormone-deprived melanoma cells (p < 0.0001; Figure 2A). These results confirm that AR also regulates melanoma proliferation in an androgen-independent manner.

To test whether *SLNCR* and AR cooperatively regulate melanoma proliferation, we introduced short, single-stranded RNA oligonucleotides designed to sterically block the interaction of *SLNCR* and AR. These oligonucleotides either mimic the *SLNCR* sequence required for AR binding and dominantly repress AR binding to *SLNCR* (mimic 1 or 2) or are the reverse complement to the *SLNCR* sequence required for AR binding, which generates double-stranded RNA incapable of binding AR (antisense 1 or 2). The antisense oligonucleotides are specifically designed to bind to *SLNCR* without eliciting RNase H-mediated degradation of *SLNCR*. Gymnotic delivery (i.e., delivery without the use of transfection reagents) of either AR-or *SLNCR*-binding 2′-deoxy-2′-fluoro-D-arabinonucleic acid (2′-FANA)-modified oligonucleotides significantly decreased melanoma proliferation without decreasing *SLNCR* expression (Figures 2B and S3D). We note that the steric blocking oligonucleotides occasionally upregulated *SLNCR* expression 2- to 3-fold, possibly resulting from a feedback loop regulating *SLNCR* expression that is initiated upon inhibition of *SLNCR* function (the mechanism of which is beyond the scope of this manuscript). Decreased cell proliferation upon inhibition of the *SLNCR*-AR interaction, despite increased *SLNCR* expression, further indicates that *SLNCR* and AR cooperatively regulate melanoma proliferation.

### *SLNCR* and AR Cooperatively Regulate Melanoma Gene Expression

**Figure 3.**
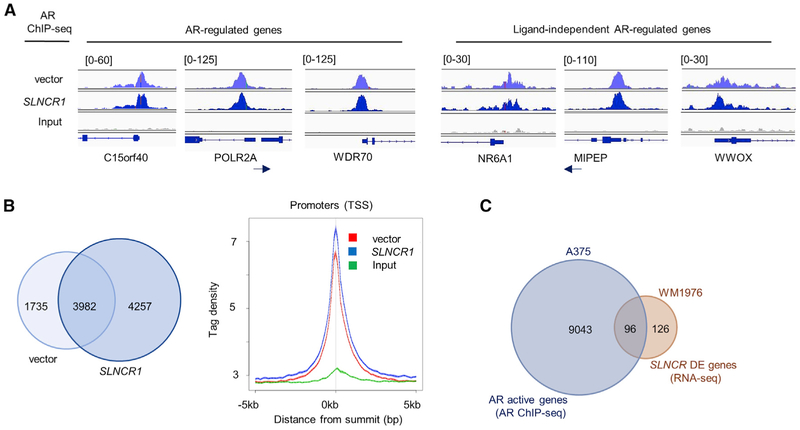
AR Binds Many *SLNCR*-Regulated Genes (A) Integrated Genome Viewer plots displaying AR ChIP-seq read intensities from vector control (light blue, top track), *SLNCR1*-expressing cells (dark blue, center track), or the input control (gray, bottom track) corresponding to the indicated genomic loci. Numbers on the top left indicate the plot height for the tracks. (B) Left panel: Venn diagram representing the numberof active genes (i.e., AR-bound genes) in A375 cells transfected with eitheran empty or *SLNCR*-expressing plasmid. Right panel: plot of tag densities for vector or *SLNCR1*-expressing cells. (C) Venn diagram representing AR active genes (as determined via AR ChIP-Seq of either vector or *SLNCR*-expressing cells), *SLNCR* differentially expressed genes (DEGs) (as determined via RNA-seq), and genes that are both AR-bound and *SLNCR*-regulated (significant enrichment, binomial test, p < 0.0001). See also Figure S4.

We next used AR chromatin immunoprecipitation and massively parallel sequencing (ChIP-seq) to identify global genomic loci bound by ligand-free AR (i.e., hormone-deprived cells). Performing AR ChIP-seq from MSTCs is technically challenging because of low AR expression; thus, we performed AR ChIP-seq from higher AR-expressing A375 melanoma cells. Because *SLNCR1* regulates AR occupancy at at least one genomic region (Schmidt et al., 2016), cells were transfected with either an empty or *SLNCR1*-expressing vector, representing either endogenous *SLNCR* levels or *SLNCR1* overexpression conditions. AR ChIP-seq identified a total of 9,974 AR binding regions (referred to as “active regions”; 5,717 for the empty vector and 8,239 for the *SLNCR1*-expressing vector) in hormone-deprived A375 melanoma cells (Table S2). The majority of the binding events in empty vector and *SLNCR1*-expressing cells occurred within 10,000 bp of annotated genes as defined by the NCBI (including both coding and non-coding, as defined by NCBI; 4,522 [78.66%] and 6,547 [79.13%] active regions, respectively; Table S2), hereafter referred to as “active genes,” suggesting that AR regulates gene expression even in the absence of canonical androgen signaling. Our AR ChIP-seq analysis faithfully identified several known AR target genes, including C15orf40, POLR2A, and WDR70 (average peak intensities, ~60–110), as well as known ligand-independent AR targets, including NR6A1, MIPEP, and WWOX (average peak intensities, ~40–175; Figure 3A) (Lin et al., 2009; Massie et al., 2007; Wang et al., 2013).

Several lines of evidence indicate that *SLNCR1* regulates AR chromatin occupancy. *SLNCR1* overexpression (1) increased the number of AR active regions (from 5,717 to 8,239 active regions with 4,257 unique sites) without increasing expression or altering localization of AR (Figure 3B; Schmidt et al., 2016), (2) increased tag density at transcriptional start sites (Figure 3B), (3) increased AR occupancy at 101 of 112 sites with altered AR binding, as identified by differential binding analysis (using model-based analysis of ChIP-seq [MACS]; Table S2), and (4) dysregulated 9 (9.2%) of the possible 98 associated genes (included in our RNA-seq analysis) exhibiting differential AR binding (p < 0.05) (Schmidt et al., 2016). Collectively, these data suggest that *SLNCR1* recruits AR to particular genomic loci. Because AR binds multiple *SLNCR1* -and *SLNCR*-regulated genes, even in the absence of ectopically expressed *SLNCR1* (Table S2), we considered all identified AR-bound genes in subsequent analyses.

To identify candidate AR- and *SLNCR*-regulated genes, we searched for genes that are both AR-bound (AR binds within 10,000 bp of gene annotation), as determined by AR ChIP-seq (Table S2), and *SLNCR*-regulated, as determined by RNA-seq (Table S1). Consistent with a functional relationship between *SLNCR* and AR, AR binding is enriched among *SLNCR*-regulated genes. For example, 25.3% of genes (9,139 of 36,074 NCBI-defined genes) were bound by AR, but 43.2% of *SLNCR*-regulated genes (96 of 222) were bound by AR (binomial test, p < 0.0001; Figure 3C; Table S2). Additionally, 45.5% of genes (50 of 110) dysregulated by *SLNCR1* overexpression were bound by AR (binomial test, p = 0.0001), and 43.8% (53 of 121) of genes dysregulated by *SLNCR1* knockdown were bound by AR (binomial test, p = 0.0003), further supporting that AR binding is enriched on *SLNCR*- and *SLNCR1*-regulated genes (Schmidt et al., 2016). Consistent with *SLNCR* and AR cooperatively regulating melanoma cell proliferation, AR binding is enriched among *SLNCR*-regulated proliferative genes (~37%, 18 of 49; Figure S4A). Interestingly, 53 AR-bound genes were upregulated upon *SLNCR* knockdown, whereas 43 genes are downregulated, suggesting that the directional effect of cooperative AR/*SLNCR* function depends on genomic context.

Many of the AR-bound, *SLNCR*-regulated genes are known or believed to play important roles in melanoma etiology, including the GRO oncogene and chemokine ligand CXCL2 (log2 fold change, −2.0), JUN oncogene (log2 fold change, 0.9), STAT3 transcription factor (log2 fold change, −0.9), interleukin-24 (IL-24; log2 fold change, 2.5), and melanoma cell adhesion molecule (MCAM; log2 fold change, 1.2). qRT-PCR of several AR-bound, *SLNCR*-regulated genes (*JUN*, *CXCL2*, and *STAT3*) before and after siRNA-mediated knockdown of either *SLNCR* or *AR* confirms that *SLNCR* and AR regulate expression of these target genes in both WM1976 and A375 cells (Figure S4B). Contrary to decreased levels upon *SLNCR* knockdown, *AR* knockdown increased the levels of *CXCL2* (1.25- to 1.75-fold), suggesting that *SLNCR* and AR may regulate the expression of certain target genes in an opposing manner. Integrative analysis of *SLNCR* RNA-seq and AR ChIP-seq datasets reveals that AR binding is enriched on *SLNCR*-regulated genes and suggest that AR and *SLNCR* similarly regulate the expression of many of these target genes both *in vitro* and *in vivo*.

Analysis of TCGA expression data reveals that AR is significantly correlated with expression of over half of *SLNCR*-regulated genes (148 of 222), 66 of which are also bound by AR based on our ChIP-seq analysis (Table S2). Correcting for multiple-hypothesis testing (Bonferroni correction) maintained the significance of AR correlation with 92 *SLNCR*-regulated genes, 43 of which are bound by AR. There is a significant concordance between target gene correlations with *SLNCR* and AR expression (Spearman r = 0.2, p = 0.003 overall), further supporting our hypothesis that *SLNCR* and AR cooperatively regulate the expression of many of these target genes *in vivo*.

### *SLNCR* and AR Cooperatively Inhibit Expression of the Cyclin-Dependent Kinase Inhibitor p21 in a p53-Independent Manner

**Figure 4.**
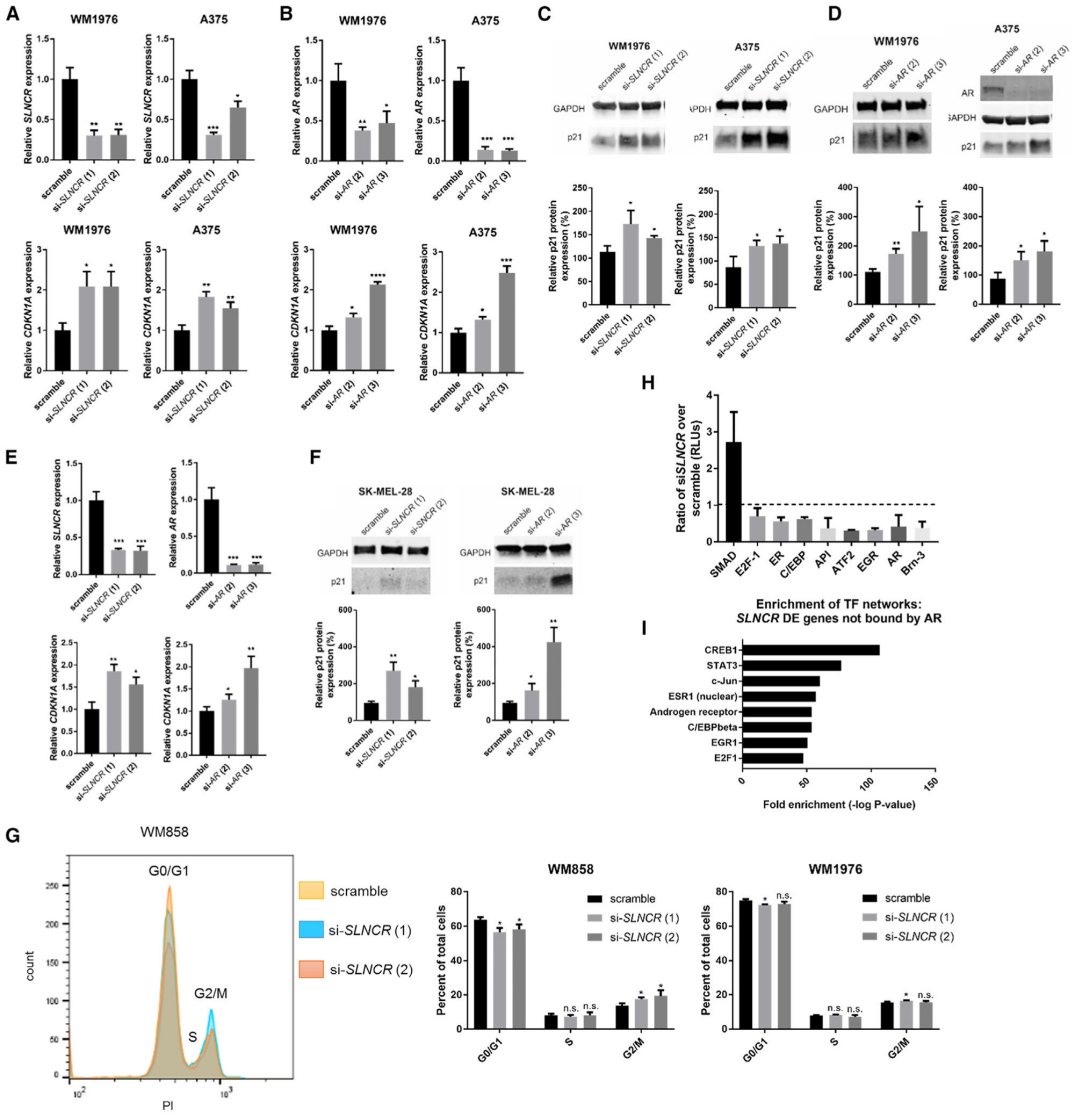
*SLNCR* and AR Inhibit Expression of *CDKN1A/*p21 Independent of p53 (A and B) Knockdown of *SLNCR* or AR increases *CDKN1A* levels. Shown is relative expression of the indicated transcripts 72 h post-transfection of the indicated cells with 10 nM of either scramble or *SLNCR*-targeting (A) or AR-targeting (B) siRNAs. qRT-PCR data are represented as the fold change compared with the scramble control, normalized to GAPDH. Error bars represent SD calculated from 3 reactions. (C and D) Knockdown of *SLNCR* (C) or AR (D) increases p21 protein levels. Protein levels were quantified using ImageJ and are presented as a fold change of p21 levels normalized to GAPDH levels. Bars represent mean ± SD from 3 independent biological replicates. (E) AR and *SLNCR* inhibit *CDKN1A* expression independent of p53. Shown is relative expression of the indicated transcripts 72 h post-transfection of the p53-deficient SK-MEL-28 melanoma cell line with 10 nM of either scramble or *SLNCR-* or AR-targeting siRNAs as in (A and B). (F) AR and *SLNCR* inhibit p21 expression independent of p53. The same as in (C) and (D), using the p53-deficient SK-MEL-28 melanoma cell line. Significance was calculated using Student’s t test: *p < 0.05, **p < 0.005, ***p < 0.0005. (G) *SLNCR* knockdown induces G2 cell cycle arrest. The cells were stained with propidium iodide (PI) 72 h post-transfection with the indicated siRNAs. Left panel: cell populations of one representative analysis. Right panels: cell populations were analyzed using FlowJo software, and significance was calculated using GraphPad Prism software. Bars represent the average percentage of total cells in the indicated stage of the cell cycle, and error bars represent SD from 3 independent replicates. Significance was calculated using a two-tailed Student’s t test via GraphPad Prism. *p < 0.05. See also Figures S1 and S2 (H) *SLNCR* regulates the activity of multiple transcription factors. Nuclear fractions were isolated from WM 1976 cells 72 h post-transfection with either scrambled or si-*SLNCR* (1) siRNA and entered directly in Signosis Transcription Factor Activation Array I. The ratio of relative luminescence units (RLUs) corresponds to the indicated transcription factor signals of si-*SLNCR* (1) versus the scramble control. Bars represent mean ± SD from 2 independent biological replicates. Shown are only transcription factors with significantly altered activity. (I) Transcription factor networks enriched among *SLNCR*-regulated genes that are not bound by AR. The analysis was performed using MetaCore (Thompson Reuters). See also Figure S5.

We next examined the mechanism of AR- and *SLNCR*-mediated regulation of one representative gene. We focused on the *SLNCR*-mediated regulation of *CDKN1A*, the gene encoding the tumor-suppressive cyclin-dependent kinase (CDK) inhibitor 1A (p21^Cip1/Waf1^), for several reasons: (1) p21 is an important regulator of cell cycle progression and anti-proliferative pathways, inducing G1 or G2 cell cycle arrest (Gire and Dulic, 2015; Giuliano et al., 2011; Yanagi et al., 2017); (2) it is commonly dysregulated in multiple tumors, including melanoma (Abbas and Dutta, 2009; Jiang et al., 1995; Vidal et al., 1995); and (3) it is transcriptionally regulated by other non-coding RNAs (Dimitrova et al., 2014; Léveillé et al., 2015; Morris et al., 2008). Additionally, p21 inhibits melanoma proliferation because depletion of p21 increases proliferation of A375 cells (Figures S4C and S4D, p < 0.0001) (Yanagi et al., 2017). In agreement with our RNA-seq analysis, knockdown of *SLNCR* in WM1976 and A375 cells significantly upregulated p21 mRNA (~1.5- to 2.5-fold increase; Figure 4A). Moreover, knockdown of *SLNCR* has been shown to increase p21 mRNA in lung cancer cells (Roth et al., 2018). AR knockdown also increased p21 mRNA levels (1.3- to 2.5-fold increase; Figure 4B). Furthermore, knockdown of either AR or *SLNCR* increased p21 protein levels (~1.4- to 3-fold; Figures 4C and 4D). Thus, *SLNCR* and AR transcriptionally repress p21 expression.

p21 can be regulated by p53-dependent mechanisms or through less well-characterized p53-independent mechanisms. Neither p53 mRNA (*TP53*) nor protein are significantly altered upon knockdown of AR or *SLNCR* in WM1976 or A375 (wild-type p53 [p53^WT^]) cells (Figures S5A-S5C). Additionally, knockdown of *SLNCR* or AR increased p21 mRNA and protein levels in the p53 mutant (p53^L145R^, inactive) primary malignant melanoma cell line SK-MEL-28 (~1.5- to 4-fold increase in both mRNA and protein; Figures 4E and 4F). These data indicate that AR and *SLNCR* repress p21 in a p53-independent manner.

We next tested whether *SLNCR* depletion mimics p21-induced melanoma phenotypes. *SLNCR* knockdown in WM858 (p53^MUT^) andWM1976 (p53^WT^) cells led to an increased percentage of cells in G2/M and a decreased percentage of cells in G1/G0 (Figure 4G; Gire and Dulic, 2015; Giuliano et al., 2011; Yanagi et al., 2017). These data demonstrate that *SLNCR* depletion phenocopies p21 induction of G2/M melanoma cell cycle arrest.

In addition to inducing cell cycle arrest, p21 binds to and regulates the activity of many transcription factors (Abbas and Dutta, 2009). We therefore quantified nuclear transcription factor binding to specific DNA motifs in WM1976 cells before and after depletion of *SLNCR* (Figures 4H and S5D). *SLNCR* knockdown reduced DNA binding of two transcription factors bound to and regulated by *SLNCR1* (AR and Brn3a) by 60%, as measured by transcription factor activation array. Decreased DNA binding by AR occurred independently of altered *AR* expression or localization (Figure S5E; Schmidt et al., 2016). *SLNCR* knockdown also decreased DNA binding by other candidate *SLNCR*-interacting proteins, including EGR1 (70%), E2F-1 (30%), ATF2 (70%), and the ATF2-containing activator protein 1 (API) transcription factor heterodimer (60%) (Schmidt et al., 2016).

*SLNCR* knockdown decreases DNA binding of known p21 targets, including the estrogen receptor (ER) and C/EBP (both ~40% activity) (Fritah et al., 2005; Harris et al., 2001), and increases DNA binding by SMAD (~270%) (Dai et al., 2012). E2F-1 is a candidate *SLNCR*-interacting protein (Schmidt et al., 2016) that is inhibited by p21 (Dimri et al., 1996; Isaeva and Mitev, 2009; Jung et al., 2010; Teplyuk et al., 2015). *SLNCR* knockdown downregulates E2F-1 DNA binding by 30%. These data suggest that *SLNCR* directly (through protein-RNA interactions) and indirectly (through p21-mediated regulation) regulates the activity of multiple transcription factors. Collectively, *SLNCR* knockdown phenocopies p21-mediated cell cycle arrest and transcription factor regulation, suggesting that *SLNCR* knockdown induces biologically relevant upregulation of p21.

Because *SLNCR* knockdown dysregulated the activity of multiple transcription factors (Figure 4H), we hypothesized that altered transcription factor activity might explain transcriptional effects of *SLNCR* not directly attributed to AR binding. When limiting our analysis to the 126 *SLNCR*-regulated genes not bound by AR (Figure 3C), we observed significant enrichment of genes within *SLNCR*-regulated transcription factor networks, as identified by the transcription factor activation array (Figure 4H), including ER, AR, C/EBP, EGR1, and E2F1 (Figure 4I). Interestingly, *SLNCR* knockdown also altered the expression of STAT3-regulated genes, a transcription factor whose expression is regulated by both *SLNCR* and AR (Table S2; Figure S4B). However, depletion of *SLNCR* does not appear to affect STAT3 activity (Figure S5D), warranting further investigation into the nature of STAT3 regulation. Collectively, these studies suggest that, in addition to cooperative transcriptional regulation of AR-bound genes, *SLNCR* regulates the expression of additional non-AR bound genes through modulation of transcription factor activity, possibly by inhibition of p21.

### *SLNCR* Recruits AR to EGR1-Bound Loci

**Figure 5.**
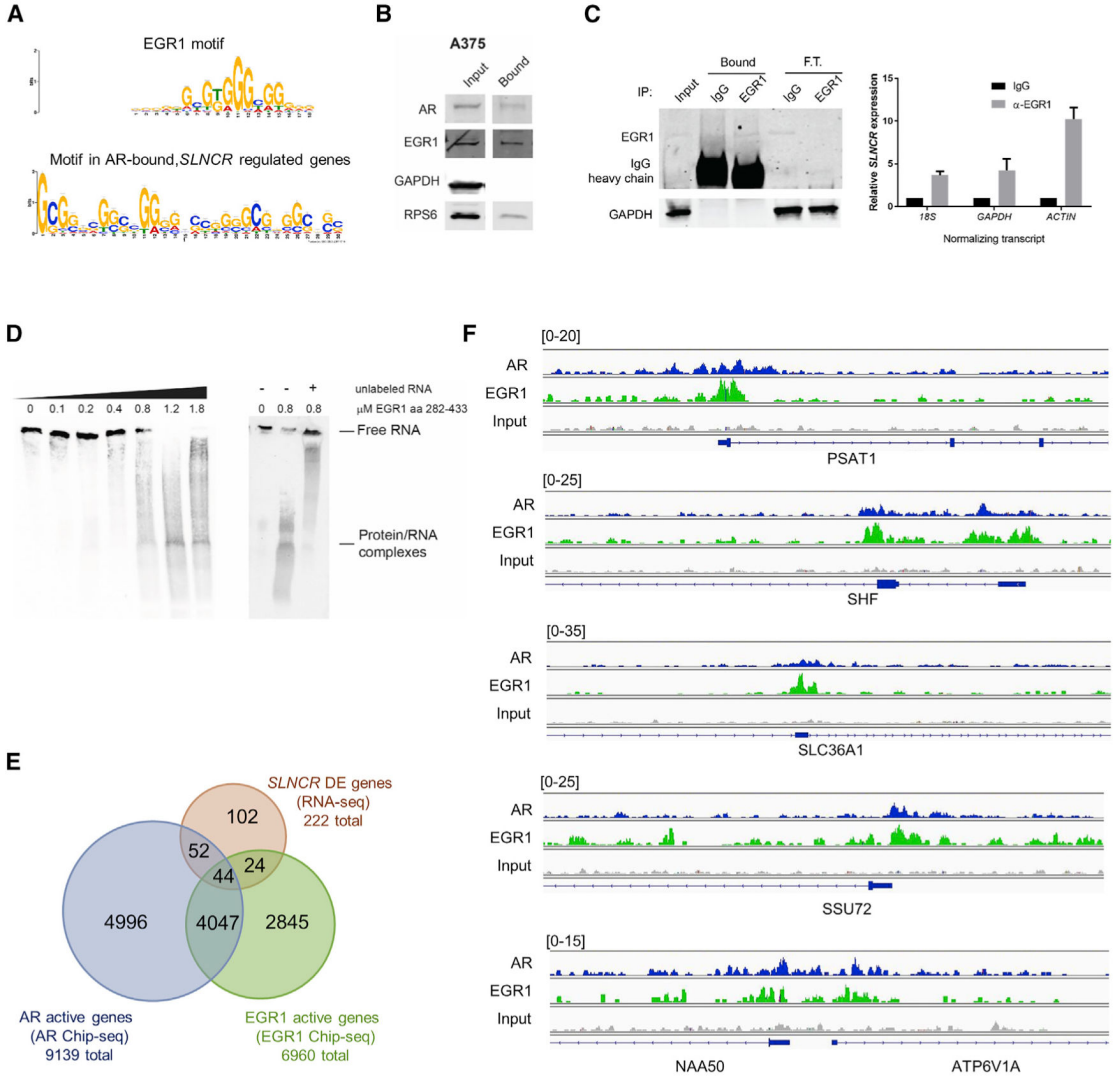
Co-binding of the *SLNCR*-Associated Transcription Factors EGR1 and AR Is Enriched on *SLNCR*-Regulated Genes (A) TOMTOM analysis of AR ChIP-seq peaks found within 10,000 bp of *SLNCR*-regulated genes(as determined via RNA-seq) identified a significant enrichment of a motif (bottom) resembling the EGR1 DNA binding motif (top) (p = 2.24e—05). (B) EGR1 is significantly enriched in the *SLNCR1* immunoprecipitate. Shown is western blot analysis of total A375 lysate (input) or immunoprecipitate enriched following incubation with *in vitro*-transcribed, biotinylated, full-length *SLNCR1* (bound). AR and the S6 ribosomal protein serves as positive controls, whereas GAPDH served as a negative control. (C) RNA immunoprecipitations from A375 cells using α-EGR1 antibody or a matched immunoglobulin G (IgG) nonspecific control. Left panel: western blot analysis of input and either bound or flowthrough (F.T.) samples following immunoprecipitation with the indicated antibody. Right panel: relative enrichment of *SLNCR* measured via qRT-PCR compared with input after normalization to the indicated transcript. (D) EGR1 binds directly to *SLNCR1.* Shown is REMSA of *in vitro*-transcribed, biotinylated, full-length *SLNCR1* following incubation with the indicated concentration of recombinant EGR1 corresponding to amino acids 282–433. Where indicated, 10 μM of unlabeled RNA competitor corresponding to full-length *SLNCR1* was added prior to addition of biotinylated *SLNCR1.* (E) Venn diagram representing genes significantly differentially expressed upon *SLNCR* knockdown (*SLNCR* DEGs) in WM1976 cells (pink) and genes bound by either AR (blue) or EGR1 (green) within 10,000 bp of an annotated gene in A375 cells (Fisher’s exact test, p < 0.0001). (F)Integrated Genome Viewer plot displaying AR (blue) and EGR1 (green) ChIP-seq read intensities for the indicated transcripts. AR ChIP-seq reads are from a sample ectopically expressing *SLNCR1.* Numbers on the top left indicate the plot height for each track. See also Figure S6.

To investigate how *SLNCR* and AR regulate gene expression, we searched for DNA sequence motifs enriched in AR ChIP-seq datasets. Multiple EM for Motif Elicitation (MEME) and TOMTOM analysis (Bailey et al., 2009) identified enrichment of the DNA binding motif of REST (or NRSF), a transcriptional repressor that regulates AR activity in prostate cancer (p = 1e–191; Figure S6A; Svensson et al., 2014). However, the REST motif was not enriched in AR binding sites among *SLNCR*-regulated genes. Instead, the DNA binding site of the EGR1 transcription factor was enriched (p = 2.24e–05; Figure 5A), suggesting that AR binds to *SLNCR*-regulated genes through a distinct mechanism, perhaps in cooperation with EGR1.

Our previous (Schmidt et al., 2016) and current (Figures 4G and 4H) work suggest that EGR1 and *SLNCR* interact directly and functionally. Incubation of biotinylated, full-length *SLNCR1* with A375 melanoma cell lysate followed by streptavidin pulldown enriched AR and EGR1 (Figure 5B). This interaction was independently validated using RNA immunoprecipitation (RIP) assays, which enriched *SLNCR* (~4- to 10-fold) in RNAs immunoprecipitating with EGR1 (Figure 5C). These data confirm that endogenous levels of *SLNCR* and EGR1 interact in A375 cells.

To distinguish between direct interaction of *SLNCR* and EGR1 versus an indirect interaction mediated by secondarily associated macromolecules, we performed RNA electrophoretic mobility shift assays (REMSAs). Recombinant EGR1 protein corresponding to amino acids 282–433 altered the mobility of full-length, *in vitro*-transcribed and biotinylated *SLNCR1* in a protein concentration-dependent manner (Figure 5D). Interestingly, EGR1 binding increased RNA mobility (sub-shifted complex), as opposed to more commonly observed decreased RNA mobility (super-shifted complex), possibly as a consequence of altered RNA secondary structure upon protein binding. Unlabeled *SLNCR1* competed for EGR1 binding, observed as a loss of increased mobility (i.e., upward shift). Collectively, these data further support the conclusion that endogenous *SLNCR* and EGR1 directly interact *in vitro* and at endogenous levels in melanoma cells.

Because *SLNCR1* binds to EGR1, and the EGR1 motif is enriched in AR-bound, *SLNCR*-regulated genes, we hypothesized that AR binds to a subset of *SLNCR*-regulated genes in cooperation with EGR1. To globally identify EGR1 binding sites in A375 cells, we performed EGR1 ChIP-seq. EGR1 binds a total of 8,373 active regions (Table S3) corresponding to a total of 6,960 active genes (Table S3). Consistent with the expected genomic occupancy, EGR1 ChIP-seq analysis identified many known EGR1-regulated genes, including *CCDC28B, ATAD2*, and the promoter of *EGR1* itself (Figure S6B; Arora et al., 2008; Kubosaki et al., 2009; Subbaramaiah et al., 2004). Unlike AR, EGR1 appears to bind its known DNA binding sequence in A375 cells because a sequence resembling this motif is the most significantly enriched in EGR1 ChIP-seq peaks (p < 1 × 10^−5^; Figure S6D).

Surprisingly, we observed a significant overlap between AR and EGR1 binding sites. Additionally, AR and EGR1 frequently co-bound at *SLNCR*-regulated genes. Although AR and EGR1 bound only 25.3% (9,139 of 36,074) and 19.3% (6,960 of 36,074) of all genes, respectively, AR bound to 58.8% of EGR1-bound genes (4,091 of 6,960 total EGR1 active genes; binomial test, p < 0.0001; Figure S6E). It is important to note that co-bound genes were identified through a stringent analysis of overlapping ChIP-seq reads. This was accomplished by directly integrating AR and EGR1 ChIP-seq reads (spanning an average of only 747 bp) rather than extrapolating binding events occurring within 10,000 bp of an annotated gene. EGR1 bound to 31% of *SLNCR*-regulated genes (68 of 222 genes; binomial test, p = 0.0003) and 46% of *SLNCR*-regulated, AR-bound genes (44 of 96 genes; Table S3; Fisher’s exact test, p< 0.0001; Figure 5E). Consistent with cooperative transcription factor binding, AR and EGR1 ChIP-seq peak read intensities overlapped within many of the 44 *SLNCR*-regulated AR- and EGR-bound genes, including *PSAT1*, *SHF*, *SLC36A11*, and *SSU72*, and the divergently transcribed *SLNCR*-regulated gene pair *NAA50* and *ATP6V1A* (Figure 5F). Collectively, these data reveal that AR and EGR binding sites overlap more frequently than expected by chance and that these sites are enriched among *SLNCR*-regulated genes. Because AR and EGR1 binding occurs at known or predicted EGR1 DNA binding motifs, these data suggest that EGR1 is required for regulation of at least a subset of AR- and *SLNCR*-regulated genes.

**Figure 6.**
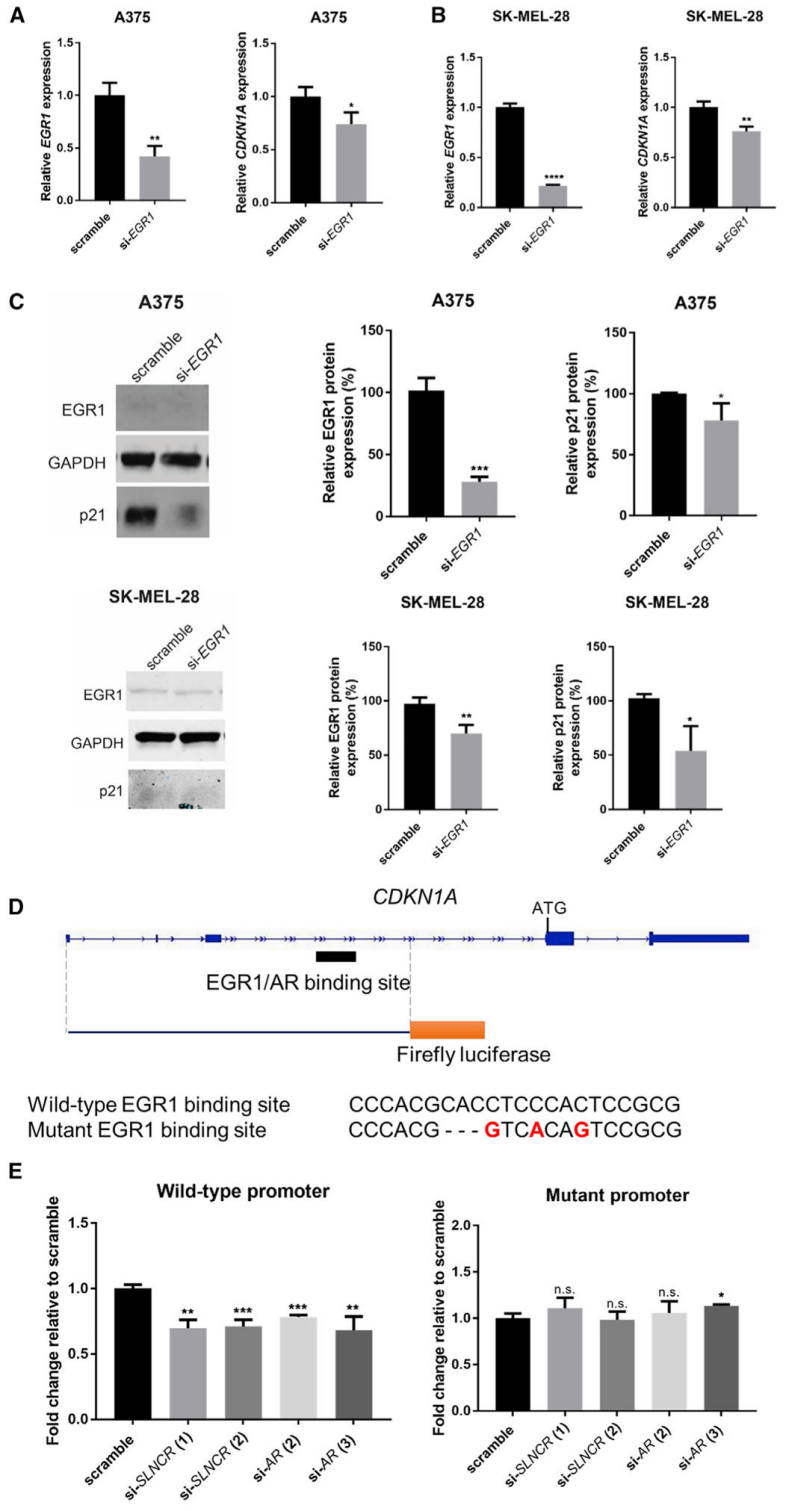
EGR1 Increases p21 Expression and Is Required for AR- and *SLNCR*-Mediated p21 Regulation (A–C) Knockdown of EGR1 decreases *CDKN1A* levels independent of p53. (A and B) Relative expression of the indicated transcripts 72 h post-transfection in (A) A375 or (B) SK-MEL-28 cells with 10 nM of either scramble or *EGR1*-targeting siRNAs. qRT-PCR data are represented as the fold change compared with the scramble control, normalized to GAPDH. Error bars represent SD calculated from 3 reactions. (C) Left panel: representative western blot analysis of A375 (top) or SK-MEL-28 (bottom) cell lysates probed for EGR1, GAPDH, or p21 levels. Center and right panels: protein levels were quantified using ImageJ and are presented as relative expression of the indicated protein, normalized to GAPDH levels. Bars represent mean ± SD from 3 independent biological replicates. (D and E) Mutation of the EGR1 DNA binding site negates AR- and *SLNCR*-mediated *CDKN1A* regulation. (D) Schematic of the *CDKN1A* locus, highlighting the sequence incorporated into the firefly luciferase reporter. The EGR1/AR binding site is denoted, with the wild-type and mutant sequences shown below, with mutated bases shown in red. (E) A375 cells were transfected with the indicated siRNAs and, 24 h later, were subsequently transfected with the wild-type (top panel) or mutant (bottom panel) *CDKN1A* firefly (FL) reporter plasmid and a CMV-RL (cytomegalovirus-*Renilla* luciferase) control. Relative FL activity was calculated as a fold change compared with vector-only control cells after normalization to RL activity. Shown is one representative assay from four independent biological replicates. Error bars represent SD from four reactions within one biological replicate. Significance was calculated using Student’s t test: *p < 0.05, **p < 0.005, ***p < 0.0005. See also Figure S7.

Because *SLNCR* binds to both AR and EGR1, and AR and EGR1 co-bind EGR1 motifs within *SLNCR*-regulated genes, we postulated that *SLNCR* recruits AR to EGR1-occupied genomic regions. If true, then EGR1 should regulate the expression of these genes, and *SLNCR*- and AR-based regulation would require an intact EGR1 DNA binding site. In support of EGR1-mediated regulation, EGR1 expression is significantly correlated with expression of over half of *SLNCR*-regulated genes (65.3%, 145 of 222), whereas significant correlation is maintained for 71 of these genes after correcting for multiple hypothesis testing (Bonferroni correction, p < 0.00023; Table S1). EGR1 positively regulates the expression of p21 because EGR1 knockdown decreased p21 mRNA and protein levels (Figures 6A-6C). Moreover, EGR1 regulates p21 independent of p53 because EGR1 knockdown in p53 mutant SK-MEL-28 cells decreased p21 levels (Figures 6B, 6C, and S7A). Thus, in contrast to *SLNCR* and AR, which repress p21, EGR1 activates p21 expression in a p53-independent manner.

To test whether *SLNCR*- and AR-mediated p21 regulation requires an intact EGR1 binding site, we generated a firefly luciferase reporter construct containing 4,663 nt of the *CDKN1A* promoter, spanning from the transcription start site to 2,966 nt upstream of the translation start codon and containing the AR- and EGR1-bound consensus EGR1 DNA binding site (Figure 6D). In contrast to regulation of the endogenous *CDKN1A* gene (Figure 4), knockdown of *SLNCR* or AR decreased expression of the ectopic *CDKN1A* luciferase reporter. This discrepancy is likely due to inherent differences between genomic and ectopically expressed plasmid DNA, which may lack the proper chromatin architecture and/or additional proximal or distant enhancer sequences required for recruitment of specific chromatin remodelers. Consistent with ligand-independent AR activation, *SLNCR* and AR knockdown also decreased expression of the *CDKN1A* reporter, even in the absence of exogenous hormones (Figure S7B). Importantly, mutation of the EGR1 binding site negated AR- or *SLNCR*-mediated regulation of the *CDKN1A* promoter, confirming that the EGR1 DNA binding site is required for *SLNCR*- and AR-based regulation of *CDKN1A*. Together, these data strongly suggest that AR and *SLNCR* associate with the *CDKN1A* promoter through DNA-bound EGR1.

### Implications of *SLNCR*-Mediated AR Activity and the Melanoma Gender Bias

The above results indicate that *SLNCR*-mediated repression of p21 requires AR and EGR1. AR has been implicated previously in the melanoma gender bias, where men suffer more frequent and severe melanomas than females (Joosse et al., 2011; Micheli et al., 2009; Siegel et al., 2015; Spanogle et al., 2010). More recent studies have confirmed oncogenic AR activity in melanoma (Schmidt et al., 2016; Wang et al., 2017). Combined with the fact that primary male melanomas express higher AR protein than female melanomas (p= 0.046; Figure S7C), it is likely that oncogenic AR activity contributes to the observed gender differences.

To explore potential contributions of *SLNCR*-mediated AR activity to these gender differences, we interrogated TCGA to determine whether p21 is expressed in a gender-specific manner. To avoid confounding our analysis with p53-dependent regulation of p21, we limited our analysis to p53-deficient melanomas (Table S4). Consistent with our model in which *SLNCR* and AR cooperatively repress *CDKN1A* expression and the fact that males express higher levels of AR, male p53-deficient melanomas express significantly lower levels of p21 than females (p = 0.045; Figure S7D). The gender-specific expression of a known melanoma tumor suppressor lends credence to the hypothesis that transcriptional regulation by *SLNCR* and AR contributes to the melanoma gender bias.

## DISCUSSION

Despite the long-held belief that AR contributes to melanomagenesis, there has been little progress in determining the role of AR in melanoma etiology. Moreover, the interpretation that AR acts as a melanoma oncogene has been confounded by the fact that AR expression is not associated with worse overall melanoma survival (data not shown). Here we comprehensively interrogated the role of AR in melanoma gene regulation, identifying many AR-regulated tumor suppressors and oncogenes. This work suggests that *SLNCR* imparts androgen-independent oncogenic activity to AR, including repression of p21. Our work highlights the importance of *SLNCR* in mediating AR’s oncogenic effects in melanoma, particularly in the context of the melanoma gender bias.

Melanoma is a complex and heterogenous disease associated with phenotypically and transcriptionally distinct growth phases. During the radial growth phase (RGP), melanomas proliferate rapidly but are unable to undergo metastasis. Upon transition to the vertical growth phase (VGP), the newly formed tumor begins to grow vertically into the dermis and acquires the ability to metastasize (Braeuer et al., 2011; Mobley et al., 2012). Moreover, melanoma cells likely fluctuate between proliferative and invasive states associated with distinct but dynamic transcriptional signatures (Hoeck et al., 2006, 2008). Although AR and *SLNCR* have been implicated previously in melanoma invasion, this work defines a role of both in the regulation of melanoma proliferation as well, suggesting that AR and *SLNCR* may be critical regulators of the RGP-to-VGP transition.

Our data suggest that inhibiting *SLNCR* function in human melanomas would decrease tumor growth and metastasis. Mouse xenografts are frequently used in preclinical development to test disease mechanisms and model therapy *in vivo*. We are planning experiments to test *SLNCR* function in a xenograft model of melanoma. In support of the observations described here, knocking down *SLNCR* decreases tumor growth and metastasis in mouse xenograft models of lung cancer and hepatocellular carcinoma (Lu et al., 2017a, 2017b; Zhang et al., 2017).

**Figure 7.**
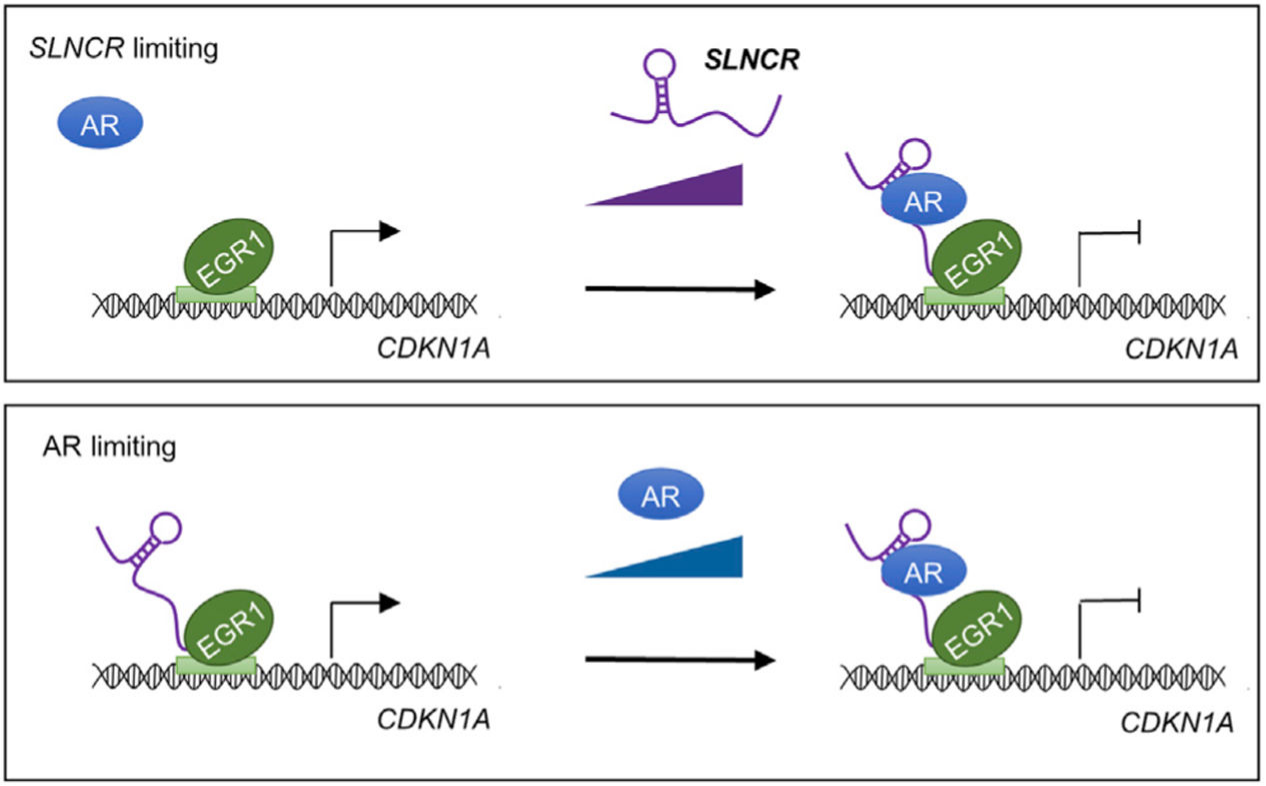
Model of AR- and *SLNCR*-Mediated, EGR1-Dependent but p53-Independent Regulation of p21 In the absence of either *SLNCR* (top panel) or AR (bottom panel), EGR1 binds to its cognate DNA binding site in the *CDKN1A* promoter and increases expression. When *SLNCR* and AR levels exceed the required threshold, *SLNCR* recruits AR to EGR1-bound chromatin to repress gene expression. See also Figure S7 and Tables S4 and S5.

Collectively, our data are consistent with a model in which *SLNCR* recruits AR to chromatin-bound EGR1 to inhibit EGR1-mediated transcriptional activation of p21 (Figure 7). Under normal physiological conditions, EGR1 binds directly to an EGR1 consensus motif located within the *CDKN1A* promoter, increasing p21 expression. These findings agree with previous reports identifying EGR1 as an important activator of p21 in glioma and gastric, colon, prostate, and breast cancer (Escoubet-Lozach et al., 2009; Kim et al., 2007, 2014; Parra et al., 2011; Shin et al., 2010,^2012^). During melanomagenesis, *SLNCR* recruits AR to chromatin-bound EGR1 to inhibit EGR1 transcriptional activation of p21. The “tethering” mechanism described here is distinct from the previously reported mechanism of *SLNCR1*-mediated regulation of *MMP9*, in which AR and Brn3a are cooperatively recruited to the *MMP9* promoter (Schmidt et al., 2016). Collectively, these works suggest that *SLNCR* can both induce novel transcriptional activity (as in the case of the cooperative recruitment of AR and Brn3a) as well as modulate preexisting regulatory networks (as for Egr1).

To further explore the relationship between p21 and known regulatory proteins and RNAs (identified here and elsewhere), we investigated possible associations between *SLNCR*, AR, EGR1, p53, and gender with p21 expression within the TCGA melanoma dataset (using available protein expression for AR, p53, and p21; n = 354). Using hierarchical multiple regression, we identified a model containing a significant three-way interaction between *EGR1* mRNA, AR, and p53 expression associated with p21 expression (estimate [95% confidence interval (CI)] = 0.12 [0.03, 0.21]; p = 0.008; Table S5). This finding is in line with data we and others have presented, indicating that EGR1 and p53 upregulate p21 (Figures 6A-6C; Alemu et al., 2011; Parra et al., 2011; Riley et al., 2008) and that AR and p53 are transcriptionally and functionally linked (Table S2; Alimirah et al., 2007; Fu et al., 2003; Kang et al., 2009; Mooslehner et al., 2012; Shenket al., 2001; Zhu et al., 2016). However, this association does not necessarily imply a direct, functional relationship between these variables, and further mechanistic studies are required to understand the biological implications of this association. Although three-way interaction models may be difficult to interpret without additional biological information, they are well suited to cope with dynamic co-expression relations and can capture complex biological associations (Bowers et al., 2004; Khayer et al., 2017). Our regression model also identified a borderline, nonsignificant three-way interaction between EGR1, AR, and *SLNCR* (estimate [95% CI] = −0.12 [−0.25, 0.001], p = 0.052) and a significant two-way interaction between *SLNCR* and EGR1 (estimate [95% CI] = −0.10 (−0.19, −0.01), p = 0.024), suggesting an inverse association between *SLNCR* and p21 expression dependent on the levels of EGR1 and AR. This is consistent with our proposed mechanism where EGR1 is required for *SLNCR*-mediated repression of p21. Considering our data indicating that (1) *SLNCR* binds EGR1 and AR (Figures 7B-7D; Schmidt et al., 2016), (2) *SLNCR* or AR knockdown increased p21 expression (Figure 4), (3) AR and EGR1 bind to an EGR1 DNA binding site in the p21 promoter, and (4) the EGR1 binding site is required for *SLNCR* and AR-mediated p21 regulation (Figures 6D and 6E), identification of this possible interaction further supports our hypothesis that *SLNCR*-mediated repression of p21 requires AR and EGR1.

According to our working model, AR is recruited to EGR1-bound loci through *SLNCR*, not through direct interaction with DNA. AR ChIP-seq peaks at *SLNCR*-regulated genes display, on average, a lower peak intensity than known AR-targets, consistent with proximal recruitment (as in our model) rather than direct DNA binding. Although current models suggest that *trans*-recruitment of lncRNA-associated chromatin modifiers occurs through triplex helix formation between the lncRNA and target DNA or through direct base-pairing between the lncRNA and proximally expressed RNAs (Davidovich and Cech, 2015), multiple lines of evidence argue against direct interaction of *SLNCR* with either DNA or nascent RNA transcripts. Specifically, (1) sequences enriched among *SLNCR*-regulated, AR-bound genes do not show significant similarity or complementarity to *SLNCR*; (2) the *CDKN1A* locus does not express proximal RNAs in the cells studied here (data not shown); and (3) knockdown of EGR1 does not phenocopy *SLNCR* knockdown (i.e., increasing p21 levels), which would be expected if *SLNCR*-mediated the EGR1 association to the *CDKN1A* promoter. Rather, our data are most consistent with a model in which the lncRNA interacts with a protein bound independently to the target loci (Davidovich and Cech, 2015). This model is strongly supported by our data indicating that *SLNCR* binds EGR1 (Figures 5B-5D) and that the EGR1 consensus DNA binding sequence in the *CDKN1A* promoter is required for *SLNCR*- and AR-mediated transcriptional regulation of *CDKN1A* (Figures 6D and 6E).

Because EGR1 is required for *SLNCR* and AR recruitment to the *CDKN1A* promoter, our working model anticipates that knockdown of EGR1 would mimic *SLNCR* and AR knockdown and increase p21 expression. However, this requires that all cells within a heterogeneous cell population express both *SLNCR* and AR in excess of EGR1-bound DNA. If not, then *SLNCR* or AR levels are limited in some cells of the heterogeneous cell population, and knockdown of EGR1 would affect normal EGR1 transcriptional upregulation (Figure 7). Indeed, the melanoma cells used in this study likely express lower levels of AR and *SLNCR* than EGR1, likely explaining why knockdown of EGR1 decreases p21 (Table S1; Figures 6A-6D; Schmidt et al., 2016).

*SLNCR*- and AR-regulated gene expression appears to be gene-specific because *SLNCR*-mediated recruitment of AR may either increase or decrease gene expression (Figure S4B). Intriguingly, *SLNCR* and AR can have opposing effects on a single locus, as seen with *CXCL2* (Figure S4B). Although our data indicate that *SLNCR* and AR are minimally required for transcriptional regulation at many of these sites, it is important to consider that other transcriptional regulators or chromatin modifiers may be recruited by *SLNCR* or that unique transcriptional regulators can be bound independently to genomic loci. These additional regulators may influence *SLNCR* and AR activity, and the presence or absence of these factors likely explains the observed complexity of *SLNCR*- and AR-mediated gene regulation. The fundamental mechanisms of gene regulation by *SLNCR* and/or AR as well as the identity of additionally recruited or precluded factors may need to be empirically determined for each individual target gene.

The observations described here provide the first global characterization of a role of AR in melanoma biology and confirm that AR binds to many melanoma-relevant genes. Remarkably, AR is associated with these regions even in the absence of canonical hormone-mediated activation, indicating that traditional antiandrogen therapies are unlikely to inhibit the oncogenic activities of AR. Instead, *SLNCR* likely recruits AR to many loci. It is important to note that *SLNCR* but not AR expression is associated with shorter overall melanoma survival (Schmidt et al., 2016) and may be required for mediating gender-specific differences in AR activity. Collectively, our data implicate *SLNCR*-mediated AR function as a novel oncogenic pathway, resulting in gender-specific differences in target gene expression. Moreover, this work is further proof that non-coding RNAs are critical regulators of human gene expression. Detailed mechanistic studies of the fundamental actions of lncRNAs and identification of their associated protein partners is critical for the design and implementation of novel therapeutic agents.

## STAR ★METHODS

### CONTACT FOR REAGENT AND RESOURCE SHARING

Further information and requests for resources and reagents should be directed to and will be fulfilled by the Lead Contact, Carl Novina (Carl_Novina@dfci.harvard.edu).

### EXPERIMENTAL MODEL AND SUBJECT DETAILS

WM1976 (p53 wild-type) and WM858 (female, BRAF^V600E^, p53^MUT^) are from the Wistar Institute collection, A375 (female, BRAF^V600E^, p53 wild-type, CDKN2A^E61^*) and SK-MEL-28 (male, BRAF^V600E^, EGFR^P753S^, P53^L145R^) cells were purchased from ATCC. The A375 and WM858 cell lines were authenticated via short tandem repeat profiling at the American Tissue Culture Repository on May 19, 2016. WM1976 and SK-MEL-28 cells were not subject to additional authentication. Unless otherwise indicated, cells were grown in DMEM (Invitrogen) without glutamine supplemented with 10% fetal bovine serum (FBS). Hormone-deprived cells were cultured in phenol-red free DMEM without glutamine (Invitrogen) with 5% charcoal-stripped FBS (Sigma-Aldrich).

### METHOD DETAILS

#### Cell culture and cell-based assays

All siRNAs were transfected using RNAiMAX (Thermo Fisher). *AR* and *SLNCR* targeting siRNAs were used at 10 nM final concentration. *EGR1* targeting siRNA was used at 20 nM. For knockdown and flutamide proliferation assays, cells were seeded at 0.4 × 10^4^ cells/well in a 96-well plate. For assays using FANA-modified oligos (AUM Technologies), cells were seeded at 0.3 × 10^4^ cells/well in a 96-well plate. Cells were treated with FANA oligos, flutamide, or transfected with the indicated siRNAs 24 hours (hr) post seeding, and proliferation was measured using a 1:10 dilution of WST-1 proliferation reagent (Roche) at the indicated time points. Cells were incubated for one hour at 37°C, and absorbance at 450 nm was measured. For cell cycle analyses, cells were harvested and washed 72 hours post-transfection, fixed in cold 70% ethanol for 2 hours, and incubated in LifeTech PI/RNaseA solution for 30 minutes at 37 degrees. For analysis of apoptosis, cells were seeded at 30 × 10^4^ cells/well in 6-well plate, harvested 72 hours post-transfection, and stained using Biolegends Pacific Blue™ Annexin V Apoptosis Kit with 7-AAD. Cells were analyzed on a Fortessa X-20 and populations were identified and quantified using FlowJo software. For luciferase assays, A375 cells were seeded in 96-well plates at 0.75 × 10^4^ cells per well, transfected 24 hours later with the indicated siRNAs. Fifty micrograms of either wild-type or mutated *CDKN1A* reporter plasmid and 50 μg of a pCMV-renilla luciferase control vector were transfected Lipofectamine® 2000 (Life Technologies) 24 hours post-transfection of siRNAs. Luciferase activity was measured another 24 hours later using Promega Dual-Glo® Luciferase Assay system. The *CDKN1A* reporter plasmid was generated by replacing the MMTV promoter in pGL4.36 vector (Promega) using Gibson Cloning (Gibson et al., 2009). Sequences for all siRNAs and oligos used in this study can be found in Table S6.

#### RNA immunoprecipitation and chromatin immunoprecipitation and sequencing

AR and EGR1 RIP assays were performed as previously described with minor modifications (Schmidt et al., 2016). For EGR1 RIP, IgG or α-EGR1 antibody was added to A375 cell lysate at a final concentration of 0.5 μg and rotated at 4°C for 2 hours. Lysate was then incubated with Protein A Dynabeads® (Life Technologies) (25 μL slurry) for 1 hour at 4°C and samples were processed as described. Fold enrichment of *SLNCR* was calculated as the fold enrichment in the IgG or EGR1 IP compared to input control after normalization to the indicated mRNA transcript (*18S*, *GAPDH*, *ACTIN*).

For AR ChIP-seq, A375 cells were cultured in phenol-red-free DMEM without glutamine (Invitrogen), supplemented with 5% charcoal-stripped FBS, and transfected with the indicated plasmid 24 hour post-seeding. For EGR1 ChIP-seq, A375 cells were cultured in DMEM without glutamine (Invitrogen), supplemented with 10% FBS, and grown to ~80% confluency. Cells were crosslinked in 1% formaldehyde for 15 min 48 hour post-transfection, and the reaction was quenched by addition of 0.125 M glycine. ChIP-seq was performed by Active Motif using Santa Cruz AR (H-280), or Cell Signaling EGR1 (44D5). After chromatin isolation and fractionation, 75-nt reads were generated by Illumina sequencing (using NextSeq 500) and were mapped to human reference genome (hg19) using the BWA algorithm with default settings. The 3′ ends of aligned reads were extended *in silico* using Active Motif software to a length of 150-200 bp. Fragment density was determined based on the number of reads corresponding to 32-nucleotide genomic bins. Peak calling, to identify intervals with local enrichment in reads, was performed using MACS (Zhang et al., 2008). MACS default cutoff p value is 1e^−7^ for narrow peaks and 1e^−1^ for broad peaks. Peak filtering was performed by removing false ChIP-Seq peaks as defined within the ENCODE blacklist. Active regions were defined by the start coordinate of the most upstream interval and the downstream coordinate of the most downstream interval. Active genes are defined as any active region present within 10,000 bps upstream or downstream of an annotated gene.

#### RNA-sequencing and analysis

Total RNA was isolated from WM1976 transfected with either scramble, si-*SLNCR* (1) or si-*SLNCR* (2) siRNAs, in duplicate, using Trizol. Sequencing cDNA libraries were prepared from 1ug of total RNA using the Illumina TruSeq RNA sample preparation kit (v2). Libraries were pooled and sequenced on the Illumina HiSeq 2500 platform. Normalized read counts (FPKM) were generated in Cufflinks v2.1.1 (http://cole-trapnell-lab.github.io/cufflinks/) by mapping onto the hg19 build of the human transcriptome (https://support.illumina.com/sequencing/sequencing_software/igenome.html). Raw FASTQ sequence was mapped using Bowtie (Langmead et al., 2009), and differentially expressed genes were identified using CuffDiff (http://cufflinks.cbcb.umd.edu/), comparing duplicate scramble controls against duplicate conditions of both *SLNCR*-specific knockdowns. Values represented in the heatmaps were generated by CuffDiff comparison of duplicate scramble controls versus duplicates of only one siRNA duplicate. GeneOntology Enrichment Analysis was performed in MetaCore (Thompson Reuters), against a control background set of genes expressed in skin cells.

Gene expression of AR- and *SLNCR*-target genes were accessed using cBioPortal (Cerami et al., 2012; Gao et al., 2013). All statistics (including t tests, ANOVAs, and correlations) were calculated using GraphPad Prism version 7.00 for Windows, GraphPad Software, La Jolla California USA (https://www.graphpad.com/). Bonferroni correction for multiple hypothesis testing was performed by defining the significance threshold as the critical p = 0.05 divided by the total number of comparisons. BAM files from RNA-seq and ChIP-seq were visualized using the Integrated Genome Viewer (https://www.broadinstitute.org/igv/) (Robinson et al., 2011; Thorvaldsdóttir et al., 2013).

#### Transcription factor activation array

WM1976 cells were seeded in 6-well tissue culture treated dishes, transfected 24 hours later with either scramble or si-*SLNCR* (1) siRNA, and were harvested and fractionated using the Thermo Scientific NE-PER Nuclear and Cytoplasmic Extraction Kit, according to the manufacturer’s instructions, 3 days after transfection. Ten micrograms of nuclear lysate was used directly as input into the Signosis TF Activation Profiling Plate Array I.

#### Protein extraction and analysis

Unless otherwise indicated, lysate was prepared using M-PER Mammalian Protein Extraction Reagent (Thermo Scientific), according to manufacturer’s instructions. Samples were separated on BioRad Any kD Mini-PROTEAN® TGX Precast Protein Gels and transferred to LF-PVDF using the mixed MW protocol on the BioRad Transblot Turbo. The following antibodies were used: Santa Cruz p53 (DO-1) sc-126 at 1:200, AR (M-20) sc-816 at 1:200, Cell Signaling P21 Waf1/Cip1 (12D1) at 1:1000, Cell Signaling EGR1 (44D5) at 1:1000, Cell Signaling S6 Ribosomal Protein (5G10) at 1:1000, and Cell Signaling GAPDH (14C10) at 1:5000.

#### RNA electrophoretic mobility shift assays

REMSAs were performed using Thermo Fisher Scientific LightShift Chemiluminescent RNA EMSA (REMSA) Kit, according to manufacturer’s instructions. Briefly, 20 μl binding reactions were assembled in low-adhesion tubes in 1X binding buffer (10mM HEPES pH 7.3, 20 mM KCl, 1 mM MgCl_2_, 1 mM DTT), with 2 μg of yeast tRNA, the indicated amount of recombinant EGR1 corresponding to amino acids 282–433 (Aviva Systems Biology, catalog number OPCD02876), 0.5 nM final concentration of the biotinylated *SLNCR1*, and 10 μM of unlabeled *SLNCR1* where indicated. Reactions were incubated at room temperature for 20 minutes, 5 μl of loading dye was added, and 20 μl was electrophoresed on Bio-Rad’s 5% Mini-PROTEAN® TBE Gel, 10 well, 30 μl. RNA and protein/RNA complexes were transferred to GE Healthcare Amersham Hybond –N+ Membrane in 0.5x TBE at 400 mA for 30 minutes in 0.5X TBE on Bio-Rad’s Trans Blot Turbo Transfer System. Detection was performed according to LightShift REMSA kit, using Bio-Rad’s ChemiDoc XRS+ System.

#### TCGA informatics and statistical analyses

GeneOntology Enrichment Analysis was performed in MetaCore (Thompson Reuters), against a control background set of genes expressed in skin cells. Gene expression of AR-and *SLNCR*-target genes, p21 protein levels, and p53 mutational status were accessed using cBioPortal (Cerami et al., 2012; Gao et al., 2013). SLNCR RNA expression values were derived from normalized read coverage across the *SLNCR* genomic range. Raw RNA-Seq data for cutaneous melanoma (SKCM) was downloaded from the NCI Genomic Data Commons (GDC). The RNA-Seq data was in the format of BAM files representing an alignment, using the STAR aligner, of raw reads to hg38. Human gene models were downloaded from RefSeq on November 3rd, 2017, and the SLNCR ranges were defined from the range of LINC00673. For each patient, the total number of reads aligning the SLNCR genomic region was obtained by parsing the output of samtools flagstat and SLNCR-specific counts were normalized by the total number of reads aligning to hg38. The expression values for non-SLNCR genes were obtained by downloading a results table from Xenabrowser (https://toil.xenahubs.net/download/tcga_rsem_isoform_tpm.gz). TPM expression values were computed from a reanalysis of the TCGA dataset under the TOIL framework using RSEM. Hierarchical multiple regression analysis was performed using R v3.2.2, where the model estimates and p values, based on the t-statistic, were calculated using the ‘lm’ function. *SLNCR* and *EGR1* mRNA expression values were log2 transformed to account for non-normal distributions. Expression of AR, p53 and p21 protein was accessed from Level 4 cross-batch normalized data in The Cancer Proteome Atlas (Li et al., 2013a). All continuous variables were converted to z-scores in order to improve interpretability of the model output. To determine the set of parameters present in the final model, standard reduction techniques were used, including iterative removal of the least significant parameter along with evaluation of the Akaike’s information criterion (AIC) and ANOVA comparisons of model fit. P53-deficient melanomas were defined as (i) primary melanomas or metastases of known melanoma origin, (ii) patients with no prior treatment, and (iii) harboring nonfunctional p53 mutations, as defined by the TP53 database (p53.fr) (Leroy et al., 2014). Patients containing R248W or Y220C gain of function p53 mutations were excluded based on reported regulation of p21 (Di Fiore et al., 2014; Song et al., 2007; Xu et al., 2014). Bonferroni correction for multiple hypothesis testing was performed by defining the significance threshold as the critical P value (0.05) divided by the total number of comparisons. BAM files from RNA-seq and ChIP-seq were visualized using the Integrated Genome Viewer (https://www.broadinstitute.org/igv/) (Robinson et al., 2011; Thorvaldsdottir et al., 2013).

### QUANTIFICATION AND STATISTICAL ANALYSIS

t tests, ANOVAs, and correlations were calculated using GraphPad Prism version 7.00 for Windows, GraphPad Software, La Jolla California USA (https://www.graphpad.com/). In proliferation assays error bars represent the mean ± SD of 3 technical replicates. Significance was calculated using the two-way analysis of variance (ANOVA), with the Dunnett test for multiple comparison testing. For binding enrichments, statistical significance was calculated using a either a Binomial test (two-sided) by comparing the observed versus expected probabilities under independence or using a Fisher Exact test of the co-bound targets, using GraphPad Prism software. In cell cycle assays, cell populations were analyzed using FlowJo software, and significance was calculated using GraphPad Prism software. Bars represent the average percent of total cells in the indicated stage of the cell cycle, and error bars represent SD from 3 independent replicates. RT-qPCR data is represented as the fold change compared to scramble control, normalized to GAPDH. Error bars represent standard deviations calculated from 3 reactions. Protein levels were quantified using ImageJ, and are presented as a fold change normalized to GAPDH levels. Bars represent mean ± SD from 3 independent biological replicates. Significance for RNA and protein quantification were calculated using the Student’s t test. Significance was calculated using a two-tailed Student’s t test. Transcription Factor Activation Array is represented by relative luminescence mean ± SD from 2 independent biological replicates.

### DATA AND SOFTWARE AVAILABILITY

The data discussed in this publication have been deposited in NCBI’s Gene Expression Omnibus (Edgar et al., 2002) and are accessible through GEO Series: GSE116191.

## Supplementary Material

1

2

3

4

5

## Figures and Tables

**Table:** KEY RESOURCES TABLE

REAGENT or RESOURCE	SOURCE	IDENTIFIER
Antibodies		
anti-AR H-280	Abcam	cat# sc-13062; RRID:AB_633881
anti-EGR1 44D5	Cell Signaling	cat# 4154; RRID:AB_591737
anti-p53 DO-1	Santa Cruz	cat# sc-126; RRID:AB_628082
anti-AR M-20	Santa Cruz	cat# sc-816; RRID:AB_1563391
anti-p21 Waf1/Cip1 12D1	Cell Signaling	cat# 2947; RRID:AB_823586
anti-EGR1 44D5	Cell Signaling	cat# 4154; RRID:AB_2097035
anti-S6 Ribosomal protein 5G10	Cell Signaling	cat# 2217; RRID:AB_331355
anti-GAPDH 14C10	Cell Signaling	cat# 2118; RRID:AB_561053
Chemicals, Peptides, and Recombinant Proteins		
recombinant EGR1 amino acids 282-433	Aviva Systems Biology	cat# OPCD02876
M-PER Mammalian Protein Extraction Reagent	Thermo FIsher Scientific	cat# 78501
WST-1 proliferation reagent	Roche, available from Sigma-Aldrich	cat# 5015944001
DMEM, high glucose, no glutamine	Invitrogen	cat# 11960044
GIBCO FBS	Thermo Fisher Scientific	cat# 26140
DMEM, high glucose, no glutamine, no phenol red	Invitrogen	cat# 31053028
Charcoal-stripped FBS	Sigma/Millipore	cat# F6765
FxCycle PI/RNase Staining Solution	LifeTech/Thermo Fisher Scientific	cat# F10797
Dynabeads Protein A for Immunoprecipitation	Invitrogen	cat# 10008D
Any kD Mini-PROTEAN^®^ TGX Precast Protein Gels	BioRad	cat# 4569033
5% Mini-PROTEAN^®^ TBE Gel, 10 well, 30 ül	BioRad	cat# 4565013
Amersham Hybond-N+	GE Healthcare Lifesciences	cat# RPN203B
Critical Commercial Assays		
LightShift Chemiluminescent RNA EMSA (REMSA) Kit	Thermo Fisher Scientific	cat# 20148
TF Activation Profiling Plate Array I	Signosis	cat# FA-1001
TruSeq RNA sample preparation kit (v2)	Illumina	cat# rs-122
Pacific Blue Annexin V Apoptosis Detection Kit with 7-AAD	Biolegend	cat# 640926
Dual-Glo® Luciferase Assay System	Promega	cat# E2920
NE-PER Nuclear and Cytoplasmic Extraction Kit	Thermo Fisher Scientific	cat# 78833
Deposited Data		
AR ChIP-seq, EGR1 ChIP-seq, WM1976 RNA-seq	This study. NCBI’s Gene Expression Omnibus	GEO: GSE116191
A375 RNA-seq	NCBI’s Gene Expression Omnibus	GEO: GSE77903
National Cancer Institute Genomic Data Commons	N/A	https://gdc.cancer.gov/
cBioPortal	Cerami et al., 2012; Gao et al., 2013	https://www.cbioportal.org/
The Cancer Proteome Atlas	Li et al., 2013a	https://tcpaportal.org/tcpa/
TP53 database	Leroy et al., 2014	http://p53.fr/
Experimental Models: Cell Lines		
WM1976 human melanoma cell line	Wistar Institute	WM1976
WM858 human melanoma cell line	Wistar Institute	WM858; RRID:CVCL_C283
A375	American Type Culture Collection	cat# ATCC^®^ CRL-1619; RRID:CVCL_0132
SK-MEL-28	American Type Culture Collection	cat# ATCC^®^ HTR-72; RRID:CVCL_0526
Oligonucleotides		
All oligonucleotides	See Table S6	N/A
Recombinant DNA		
pGL4.36[luc2P/MMTV/Hygro] Vector, 20ug	Promega	cat# E1360
Software and Algorithms		
FlowJo Software	FlowJo	https://www.flowjo.com/
MACS	Zhang et al., 2008	http://liulab.dfci.harvard.edu/MACS/
Cufflinks v2.1.1	Trapnell et al., 2012	http://cole-trapnell-lab.github.io/cufflinks/
MetaCore	Thompson Reuters, now Clarivate	https://clarivate.com/products/metacore/
GraphPad Prism version 7.00 for Windows	GraphPad Software	https://www.graphpad.com/
Integrated Genome Viewer	Robinson et al., 2011; Thorvaldsdottir etal., 2013	https://www.broadinstitute.org/igv/

## Highlights

- SLNCR-AR complexes drive melanoma growth and invasion
- SLNCR-AR complexes are recruited to EGR1-bound loci
- SLCNR-AR-EGR1 complexes regulate transcription of proliferative genes
- EGR1 normally activates p21; SLNCR-AR-EGR1 complexes repress p21
