# Supplementary material for: The lncRNA SLNCR Recruits the Androgen Receptor to EGR1-Bound Genes in Melanoma and Inhibits Expression of Tumor Suppressor p21
Source: Cell Rep. Author manuscript; Available in PMC 2019 Jul 31. (PMC6668037; doi:10.1016/j.celrep.2019.04.101)
Supplement: 1 [file NIHMS1530019-supplement-1.pdf]

Cell Reports, Volume *27*

Supplemental Information

The lncRNA *SLNCR* Recruits the Androgen Receptor to EGR1-Bound Genes in Melanoma and Inhibits Expression of Tumor Suppressor p21

Karyn Schmidt, Johanna S. Carroll, Elaine Yee, Dolly D. Thomas, Leon Wert-Lamas, Steven C. Neier, Gloria Sheynkman, Justin Ritz, and Carl D. Novina

SUPPLEMENTAL FIGURES AND LEGENDS

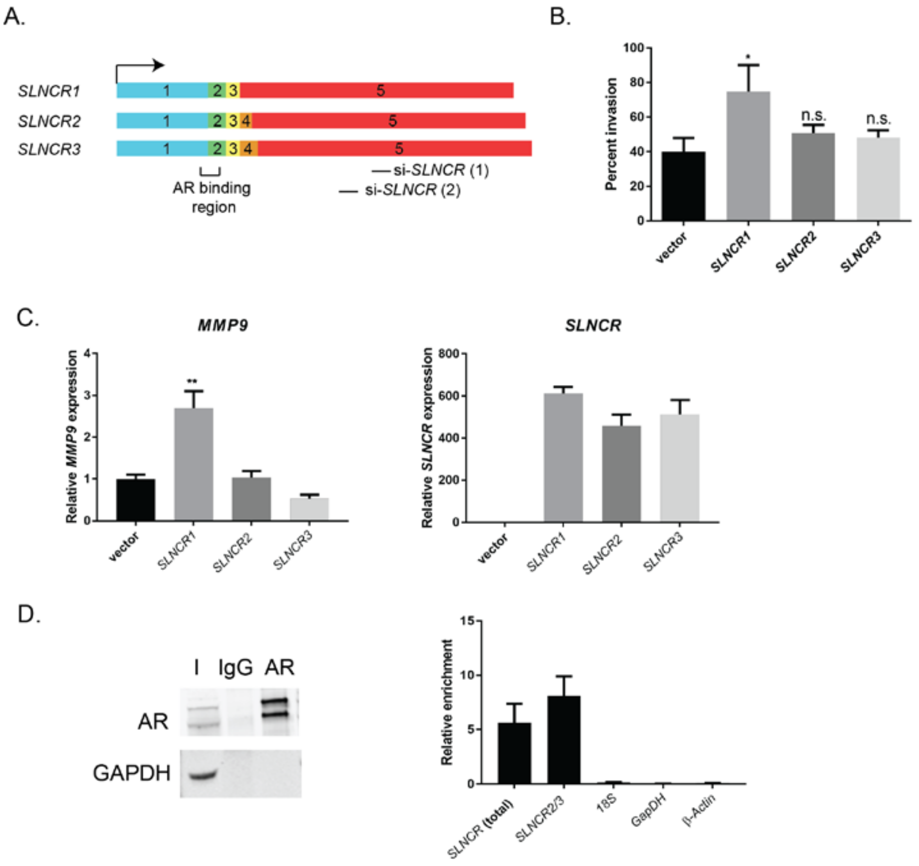

Figure S1: *SLNCR1*, but not *SLNCR2* nor *SLNCR3*, increase melanoma invasion, related to Figure 1. (A) Schematic of the exons (numbered) of the 3 *SLNCR* isoforms previously identified in melanomas (not drawn to scale). Denoted is the sequence required for AR binding, and the regions targeted by the siRNAs used in this study. (B) Quantification from 3 independent replicates, represented as mean ± SD, of matrigel invasion assays of A375 cells transfected with the indicated empty or *SLNCR*-expressing plasmid. Invasion is calculated as the percent of invading cells compared to mobile cells as counted in 8 fields of view. (C) Relative *MMP9* (left) or *SLNCR* (right) expression in A375 cells transfected with the indicated empty or *SLNCR*-expressing plasmid. RT-qPCR data is represented as the fold change compared to scramble control, normalized to *GAPDH*. Error bars represent standard deviations calculated from 3 reactions. Significance was calculated using the two-tailed Student's t-test: ** $p < 0.005$. (D) RNA immunoprecipitations from HEK293T cells transfected with GFP-tagged AR, using α-AR antibody or a matched IgG nonspecific control. Left panel: western blot analysis of input or bound proteins following IP with either IgG or α-AR (AR) antibodies. Right panel: relative enrichment of the indicated transcript measured via RT-qPCR compared to IgG nonspecific control.

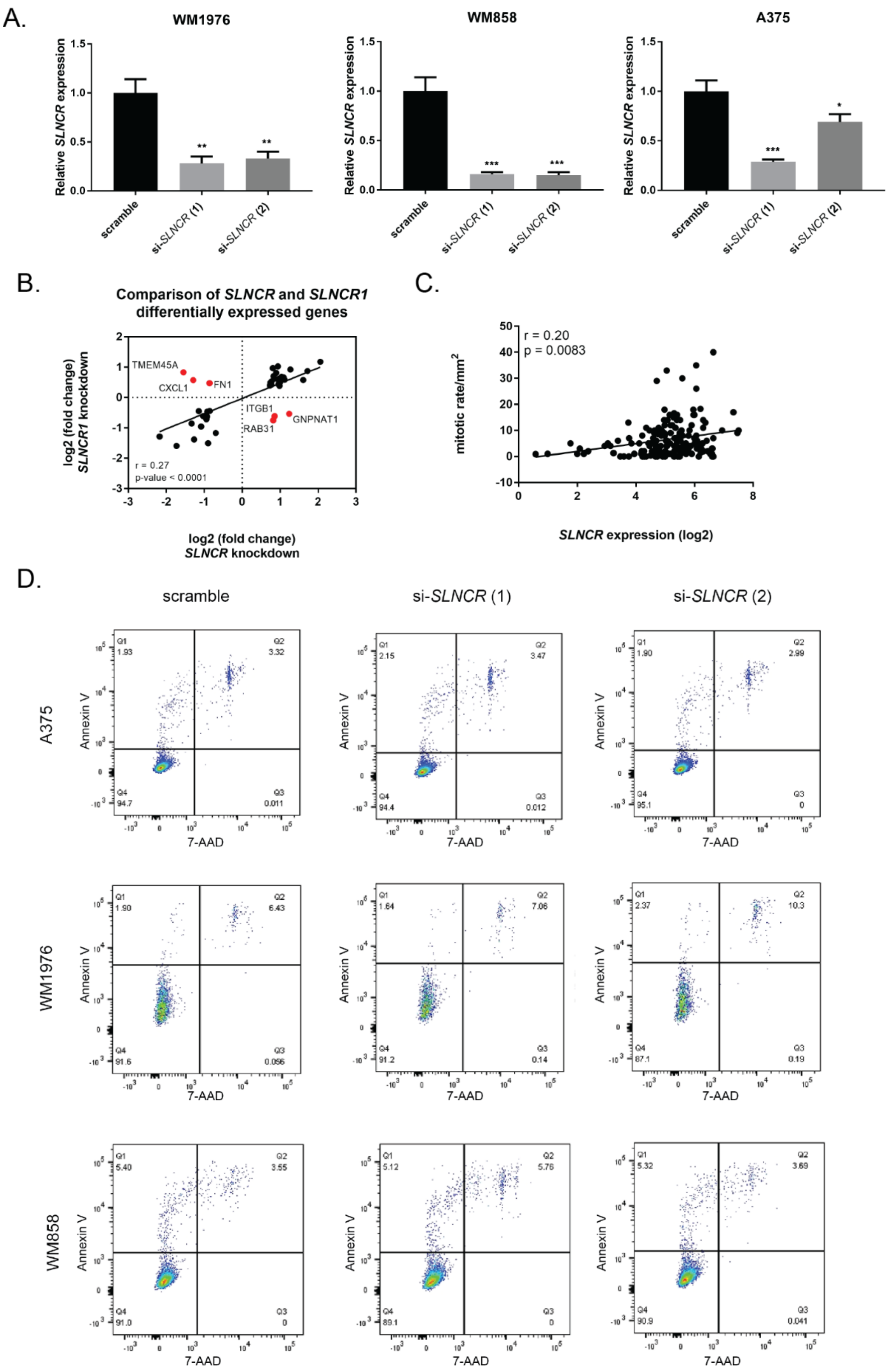

Figure S2: siRNA-mediated knockdown of *SLNCR* does not affect melanoma apoptosis, related to Figure 1. (A) Relative *SLNCR* expression in the indicated cells transfected with either scramble or *SLNCR*-targeting siRNAs.

RT-qPCR data is represented as the fold change compared to scramble control, normalized to *GAPDH*. Error bars represent standard deviations calculated from 3 reactions. Significance was calculated using the two-tailed Student's t-test: * $p < 0.05$, ** $p < 0.005$, *** $p < 0.0005$, n.s. = not significance. (B) Scatter plot of the log2 fold change of genes significantly dysregulated ($p < 0.01$) upon knockdown of both *SLNCR1* and *SLNCR* (all isoforms). Genes that are dysregulated in an opposing manner by *SLNCR1* versus *SLNCR* are labeled and denoted in red. Pearson's correlation (r) was calculated using GraphPad Prism. (C) Scatter plot of primary melanoma mitotic growth rate versus Log2 *SLNCR* expression for 172 melanomas from the TCGA (mitotic growth rate was available for only 172 melanomas). (D) Representative scatter plots of annexin V and 7-AAD staining, as measured via fluorescence-activated cell sorting (FACS) analysis, of the indicated melanoma cells transfected with the indicated siRNAs. Cells are classified as "viable" (Q4, bottom left), "apoptotic" (Q1, top left), or "necrotic" (Q4, top right).

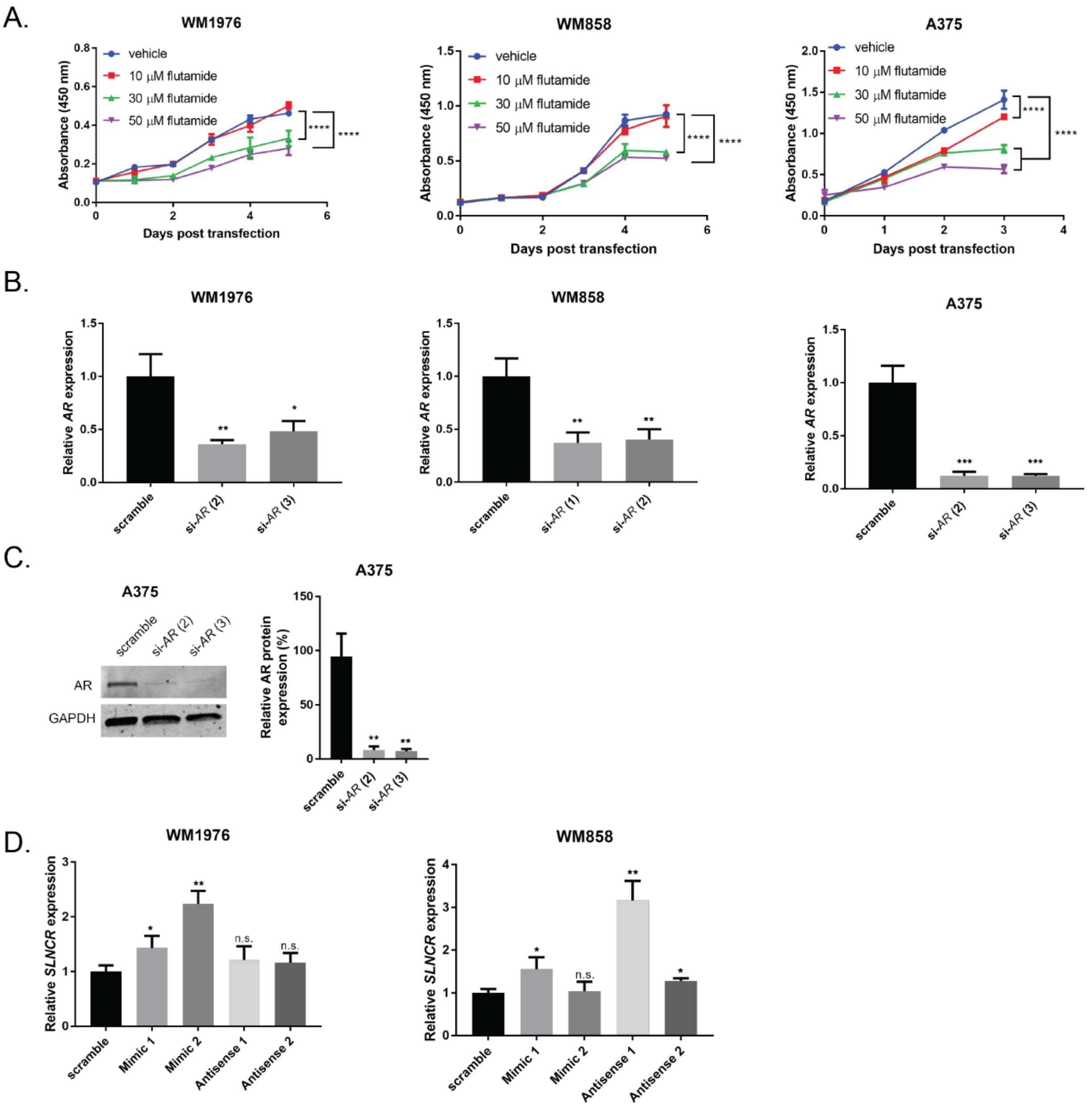

Figure S3: AR increases melanoma cell proliferation, related to Figure 2. Twenty-four hours after the indicated cells were seeded in 96-well plates, vehicle-control or the indicated concentration of flutamide was added. Cell proliferation was quantified using WST-1 reagent, as in Figure 1D. (B) Relative AR expression in the indicated cells transfected with either scramble or *SLNCR*-targeting siRNAs. (C) Western blot of A375 cell lysates following transfection with the indicated siRNAs. Left panel: representative blot probed with α-AR and α-GAPDH antibodies. Right panel: quantification from three independent replicates, normalized to GAPDH. (D) Relative *SLNCR* expression in the indicated cells following addition of the indicated FANA-modified oligos. RT-qPCR data ((A) and (C)) is represented as the fold change compared to scramble control, normalized to GAPDH. Error bars represent standard deviations calculated from 3 reactions. Significance was calculated using the Student's t-test: * $p < 0.05$, ** $p < 0.005$, *** $p < 0.0005$.

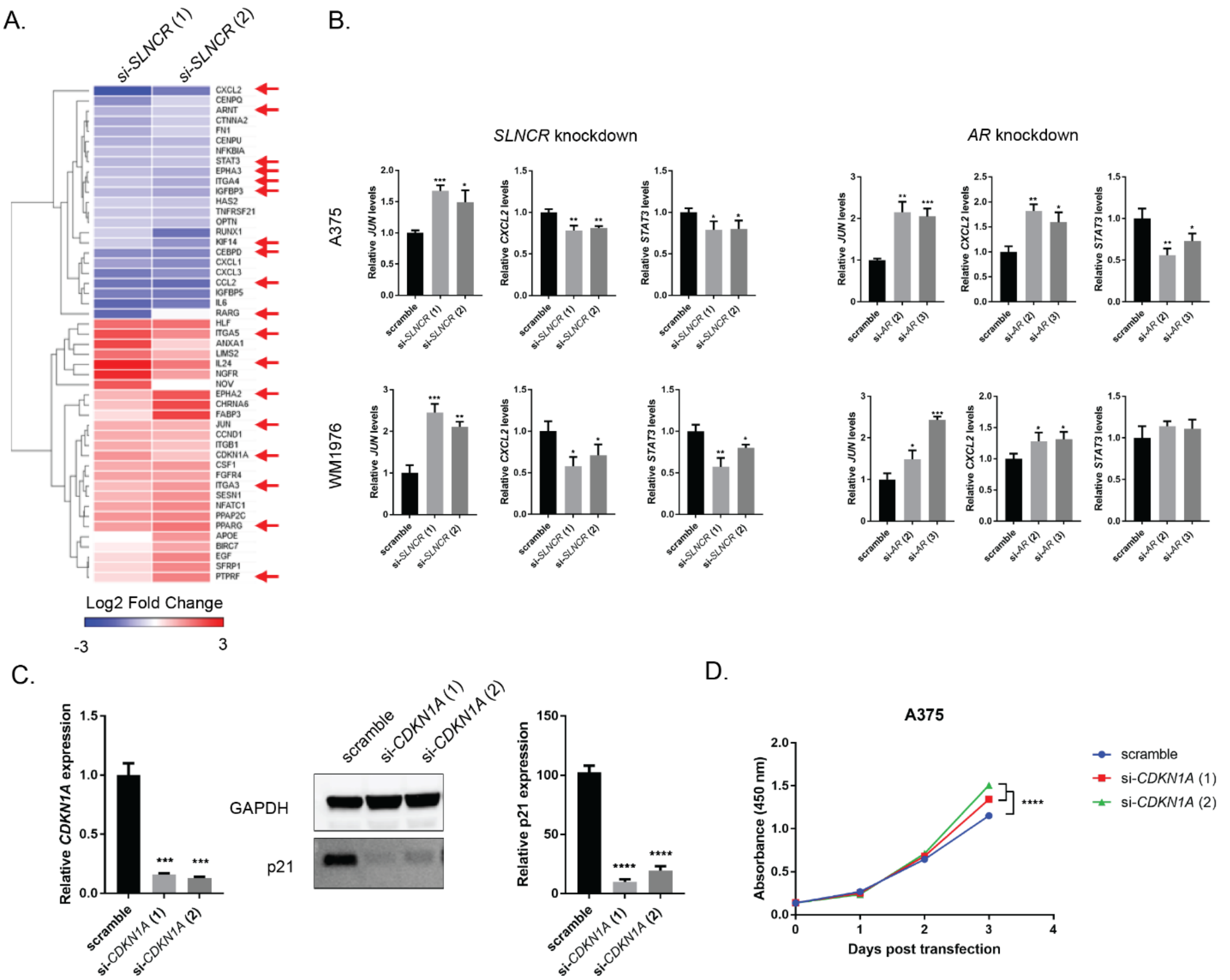

Figure S4: *SLNCR* and AR regulate many overlapping genes, including genes implicated in melanoma proliferation, related to Figure 3. (A) Heat map of *SLNCR*-regulated genes (as in Figure 1A) that are also AR-bound. The shading represents the log2 fold change compared to scramble siRNA control. Genes are clustered with Euclidean distance and average linkage clustering. Red arrows denote genes implicated in cell proliferation. (B) Relative expression of the indicated genes from indicated cells transfected with either scramble, *SLNCR*-, or AR-targeting siRNAs. (C) Knockdown of *CDKN1A* in A375 cells. Left panel: Relative *CDKN1A* expression following transfection of the indicated siRNAs, represented as the fold change compared to scramble control, normalized to GAPDH. Middle panel: western blot of A375 cell lysates following transfection with the indicated siRNAs probed with α-p21 and α-GAPDH antibodies. Right panel: quantification from three independent replicates, normalized to GAPDH. (D) Cell proliferation was quantified using WST-1 reagent following transfection with the indicated siRNAs. Significance was calculated using the two-way analysis of variance (ANOVA), with the Dunnett test for multiple comparison testing, **** $p < 0.0001$. RT-qPCR data is represented as the fold change compared to scramble control, normalized to GAPDH. Error bars represent standard deviations calculated from 3 reactions. Significance was calculated using the Student's t-test: * $p < 0.05$, ** $p < 0.005$, *** $p < 0.0005$.

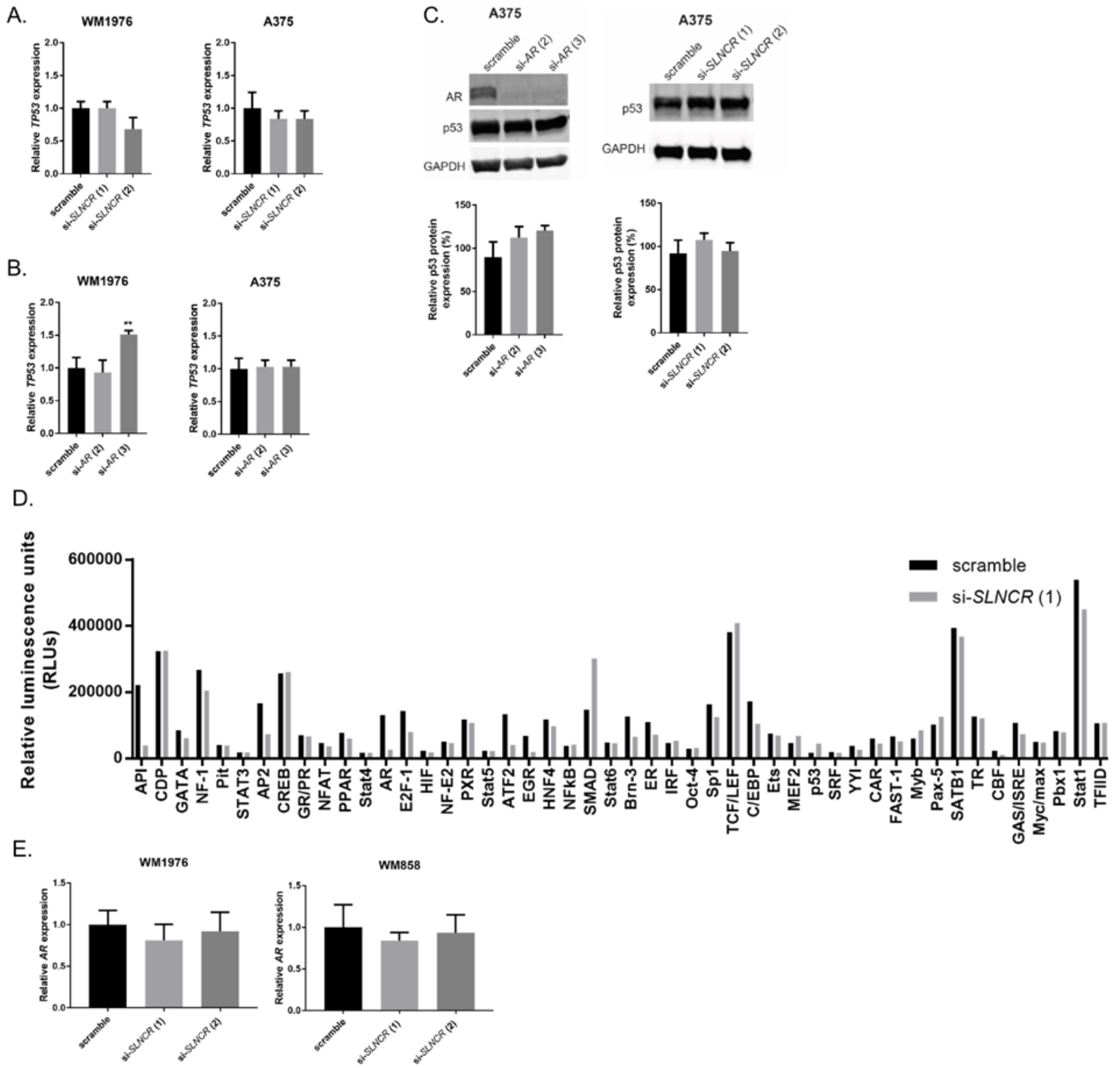

Figure S5: *SLNCR* and AR do not directly regulate expression of p53, related to Figure 4. (A and B) Relative expression of *TP53* 72 hours post-transfection of the indicated cells with 10 nM of either scramble or *SLNCR* (A) or *AR* (B) targeting siRNAs. (C) Knockdown of *SLNCR* or AR in A375 melanoma cells does not affect p53 expression. Protein levels were quantified using ImageJ, and are presented as a fold change of p21 levels, normalized to GAPDH levels. Bars represent mean ±SD from 3 independent biological replicates. (D) Transcription factor activity in WM1976 nuclear lysate following transfection with either scramble or si-*SLNCR* (1) siRNA was measured by Signosis' Transcription Factor Activation Array I. The raw relative luminescence units (RLUs) are shown for all transcription factor probes included in the array. (E) Knockdown of *SLNCR* does not affect *AR* expression. Relative expression of *AR* 72 hours post-transfection of the indicated cells with 10 nM of either scramble or *SLNCR* targeting siRNAs. RT-qPCR data is represented as the fold change compared to scramble control, normalized to GAPDH. Error bars represent standard deviations calculated from 3 reactions. Significance was calculated using the Student's t-test and is only indicated where determined to be significant: ** $p < 0.005$.

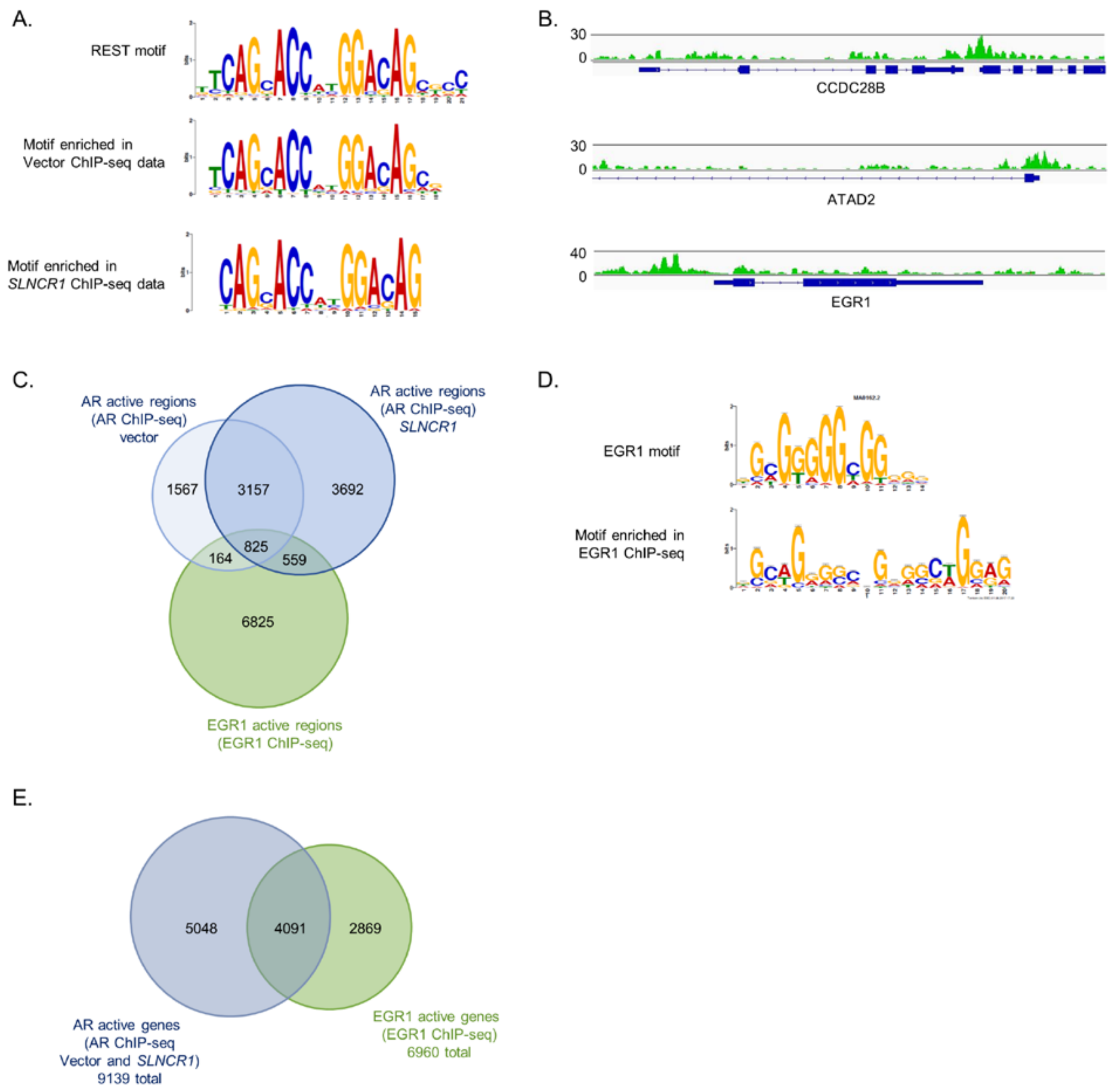

Figure S6. AR and EGR1 cobind EGR1 consensus DNA motifs in melanoma cells, related to Figure 5. (A) A motif resembling the consensus REST DNA binding motif (top panel) is significantly enrichment in AR ChIP-seq peaks with vector (middle) or SLNCR-expression plasmid (bottom). (B) Integrated Genome Viewer plot displaying EGR1 ChIP-seq read intensities for the indicated transcripts. Numbers on the left indicated plot height. (C) Venn diagram representing the total regions bound by either AR in A375 cells transfected with vector (light blue) or *SLNCR1*-expressing plasmid (blue), or bound by EGR1 (green). (D) TOMTOM analysis identified a significant enrichment of a motif in EGR1 ChIP-seq peaks (bottom) showing significant similarity to the consensus EGR1 DNA binding motif (top panel). (E) Venn diagram representing the genes bound by either AR (blue) or EGR1 (green) within 10,000 bp of an annotated gene in A375 cells.

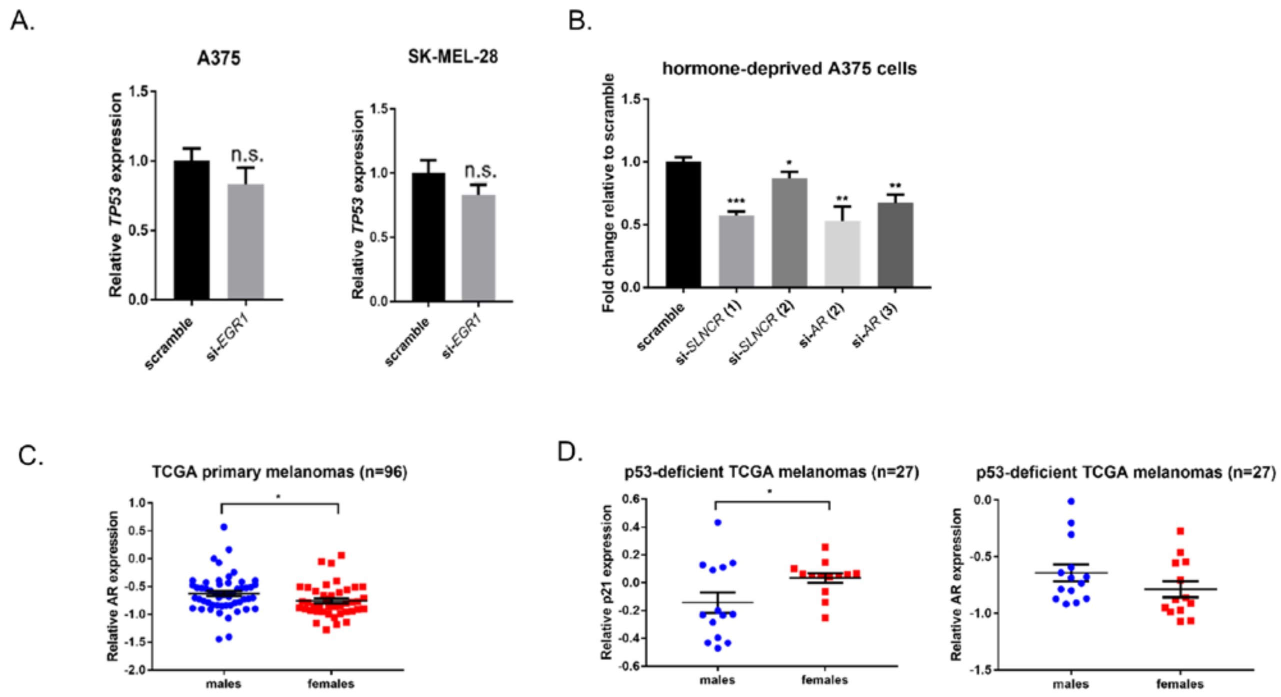

Figure S7: AR and *SLNCR*-mediated regulation of p21 does not require p53 or androgens and occurs in a gender-specific manner, related to Figures 6 and 7. (A) Knockdown of EGR1 does not affect TP53 levels. Relative expression of TP53 72 hours post-transfection of either A375 (left) or SK-MEL-28 (right) cells with 10 nM of either scramble or EGR1-targeting siRNAs. RT-qPCR data is represented as the fold change compared to scramble control, normalized to GAPDH. Error bars represent standard deviations calculated from 3 reactions. (B) *SLNCR* and AR regulate the CDKN1A promoter in the absence of androgens. Same as in Figure 5D, using A375 cells grown in hormone-starved conditions. (C) Box plot of relative AR protein expression, determined via reverse phase protein array (RPPA), in male and female TCGA primary melanomas. Data are represented as mean ± SEM. (D) Box plot of relative p21 (left) and AR (right) expression but for only p53-deficient melanomas. Significance was calculated using the Student's t-test: * $p < 0.05$, ** $p < 0.005$, *** $p < 0.0005$, ns = not significant.

PATIENT ID	Gender	TP53 mutational status	P21 (RPPA)	AR (RPPA)
TCGA-EE-A29M	FEMALE	F134L	0.14587	-0.86133
TCGA-DA-A1HW	FEMALE	K120E	0.040554	-0.9723
TCGA-GF-A6C8	FEMALE	P278S	0.052421	-0.5473
TCGA-BF-A3DL	FEMALE	R196*	0.065464	-0.95115
TCGA-DA-A1I5	FEMALE	R196*, E286K	0.052141	-0.98755
TCGA-EB-A5FP	FEMALE	R213*	0.25405	-0.55538
TCGA-EE-A3AE	FEMALE	R213*	-0.063335	-1.0698
TCGA-DA-A1HV	FEMALE	R280K	0.062088	-0.70658
TCGA-EE-A181	FEMALE	V143E	0.053279	-0.91168
TCGA-EE-A2MM	FEMALE	V143G	-0.25283	-0.46138
TCGA-ER-A199	FEMALE	X126_splice	-0.13843	-0.87842
TCGA-D3-A51T	FEMALE	X187_splice	0.10025	-1.0643
TCGA-ER-A19E	FEMALE	X224_splice	0.059999	-0.27543
TCGA-FS-A4F9	MALE	A276Lfs*29	-0.47049	-0.67793
TCGA-EB-A3XC	MALE	C275Y	0.11002	-0.78656
TCGA-EE-A2MU	MALE	G266E	0.4328	-0.011376
TCGA-D3-A1QA	MALE	H214Qfs*7	-0.28437	-0.20244
TCGA-EE-A29E	MALE	L330R	-0.2328	-0.87094
TCGA-EE-A3AA	MALE	P151L, P27S, P151S	0.12717	-0.73382
TCGA-EE-A29L	MALE	P177_C182del	-0.22706	-0.9179
TCGA-EE-A2GC	MALE	P177L	-0.43232	-0.68729
TCGA-GN-A266	MALE	R290C, A159V	0.090022	-0.30487
TCGA-GF-A6C9	MALE	S127F	-0.43199	-0.79911
TCGA-EE-A3AD	MALE	S241F	-0.23497	-0.9065
TCGA-EE-A3J7	MALE	S241F	-0.20028	-0.6418
TCGA-FS-A1Z4	MALE	V97Sfs*26	0.1418	-0.59557
TCGA-D9-A6EC	MALE	X187_splice, R213*	-0.39416	-0.8732

Table S4: AR and p21 protein expression of p53-deficient TCGA melanomas, related to Figure 7. P53-deficient melanomas were defined as (i) primary melanomas or metastases of known melanoma origin, (ii) patients with no prior treatment, and (iii) harboring nonfunctional p53 mutations, as defined by the TP53 database (p53.fr) (Leroy et al., 2014). Three additional patients containing R248W or Y220C gain of function p53 mutations were excluded based on reported regulation of p21 (Di Fiore et al., 2014; Song et al., 2007; Xu et al., 2014).

Variable or interaction	Estimate (95% CI)	p-value
Intercept	-0.05 (-0.15, 0.06)	0.38
SLNCR	-0.07 (-0.17, 0.03)	0.19
EGR1 mRNA	-0.12 (-0.23, -0.02)	0.023
AR protein	0.04 (-0.07, 0.15)	0.49
P53 protein	-0.13 (-0.24, -0.01)	0.027
SLNCR * EGR1	-0.10 (-0.19, -0.01)	0.024
SLNCR * AR	0.002 (-0.11, 0.11)	0.96
EGR1 * AR	-0.05 (-0.16, 0.06)	0.34
EGR1 * p53	<0.001 (-0.11, 0.11)	0.99
AR * p53	0.12 (0.05, 0.18)	0.0004
EGR1 * AR * SLNCR	-0.12 (-0.25, 0.001)	0.052
EGR1* AR * p53	0.12 (0.03, 0.21)	0.008

Table S5: Final model determined from hierarchical multiple regression analysis of p21 protein expression in TCGA melanomas (n=354), related to Figure 7. Adjusted R-squared = 0.06, Model (F-statistic) p = 0.0009. Variables assessed as part of the multiple regression analysis of p21 expression included patient gender, *SLNCR* and *EGR1* mRNA log2 expression, and AR and p53 protein expression (from TCPA) and all possible interactions. All continuous variables were converted to z-scores in order to improve interpretability of the model output. In order to determine the set of parameters present in the final model, standard reduction techniques were used, including iterative removal of the least significant parameter along with evaluation of the Akaike's information criterion (AIC) and ANOVA comparisons of model fit. Non-significant parameters remain in the final model due to the presence of significant two- and three-way interactions. P-values have not been adjusted for multiple hypothesis testing.

Sequence	Name	Type
TTAGGTCAAATAGGATCTAAA (targeting)	si-*SLNCR* (1)	siRNA
AAAGACGTTTACACCGAGAAA (targeting)	si-*SLNCR* (2)	siRNA
CAGGAATTCCTGTGCATGAAA (targeting)	si-*AR* (1)	siRNA
CACGGGAAGTTTAGAGAGCTA (targeting)	si-*AR* (2)	siRNA
CTGCTACTCTTCAGCATTATT (targeting)	si-*AR* (3)	siRNA
GAAGGTGAAGGTCGGAGT	*GAPDH* Forward	qPCR
GAAGATGGTGATGGGATTTC	*GAPDH* Reverse	qPCR
GTGGAGCGATTTGTCTGGTT	*18S* Forward	qPCR
CGCTGAGCCAGTCAGTGTAG	*18S* Reverse	qPCR
AACTTCTACAATGAGCTGCG	*β-ACTIN* Forward	qPCR
CCTGGATAGCAACGTACATGG	*β-ACTIN* Reverse	qPCR
GAGAACGTGGTGGAATCAGA	*SLNCR* Forward (all isoforms)	qPCR
TCCCATCCTCTTTCTTGTCC	*SLNCR* Reverse (all isoforms)	qPCR
GGTTACACCAAAGGGCTAGAA	*AR* Forward	qPCR
GACTTGTAGAGAGACAGGGTAGA	*AR* Reverse	qPCR
TGTCACTGTCTTGTACCCTTG	*CDKN1A* Forward	qPCR
GGCGTTTGGAGTGGTAGAA	*CDKN1A* Reverse	qPCR
GCCATCTACAAGCAGTCACAG	*TP53* Forward	qPCR
TCATCCAAATACTCCACACGC	*TP53* Reverse	qPCR
GTGTGTCTCCGCTTGAAGCTTGGCAATCCGGTAC	Remove MMTV from pGL4.36	Gibson cloning primer
CTCTTCTATGCCAGGGCCAGTTAGGCCAGAGAAATGTTC	Remove MMTV from pGL4.36	Gibson cloning primer
CTGGCATAGAAGAGGCTGGT	*CDKN1A* promoter Foward	PCR amplification
CAAGCGGAGACACACTGGTAT	*CDKN1A* promoter Reverse	PCR amplification
GTCACAGTCCGCGCGTGATTTCCTGAG	EGR1 binding site mutant	Site-directed mutagenesis
GTGCGTGGGCAGAGCCTCGGAC	EGR1 binding site mutant	Site-directed mutagenesis

Table S6: siRNA targets and oligo sequences used in this study, related to Figures 1-7. Red font denotes mutated nucleotides.
